# Author Correction: Heritability estimates for 361 blood metabolites across 40 genome-wide association studies

**DOI:** 10.1038/s41467-020-15276-y

**Published:** 2020-03-31

**Authors:** Fiona A. Hagenbeek, René Pool, Jenny van Dongen, H. M. Draisma, Jouke Jan Hottenga, Gonneke Willemsen, Abdel Abdellaoui, Iryna O. Fedko, Anouk den Braber, Pieter Jelle Visser, Eco J. C. N. de Geus, Ko Willems van Dijk, Aswin Verhoeven, H. Eka Suchiman, Marian Beekman, P. Eline Slagboom, Cornelia M. van Duijn, BBMRI Metabolomics Consortium, J. J. H. Barkey Wolf, D. Cats, N. Amin, J. W. Beulens, J. A. van der Bom, N. Bomer, A. Demirkan, J. A. van Hilten, J. M. T. A. Meessen, M. H. Moed, J. Fu, G. L. J. Onderwater, F. Rutters, C. So-Osman, W. M. van der Flier, A. A. W. A. van der Heijden, A. van der Spek, F. W. Asselbergs, E. Boersma, P. M. Elders, J. M. Geleijnse, M. A. Ikram, M. Kloppenburg, I. Meulenbelt, S. P. Mooijaart, R. G. H. H. Nelissen, M. G. Netea, B. W. J. H. Penninx, C. D. A. Stehouwer, C. E. Teunissen, G. M. Terwindt, L. M. ‘t Hart, A. M. J. M. van den Maagdenberg, P. van der Harst, I. C. C. van der Horst, C. J. H. van der Kallen, M. M. J. van Greevenbroek, W. E. van Spil, C. Wijmenga, A. H. Zwinderman, A. Zhernikova, J. W. Jukema, H. Mei, M. Slofstra, M. Swertz, E. B. van den Akker, J. Deelen, M. J. T. Reinders, Amy C. Harms, Thomas Hankemeier, Meike Bartels, Michel G. Nivard, Dorret I. Boomsma

**Affiliations:** 1 Department of Biological Psychology, Vrije Universiteit Amsterdam, Amsterdam, The Netherlands; 2 Amsterdam Public Health Research Institute, Amsterdam, The Netherlands; 3 Alzheimer Center Amsterdam, Department of Neurology, VU Amsterdam, Amsterdam UMC, Amsterdam, The Netherlands; 4 Amsterdam Neuroscience, Amsterdam, The Netherlands; 5 Department of Psychiatry and Neuropsychology, School of Mental Health and Neuroscience, Alzheimer Center Limburg, Maastricht University, Maastricht, The Netherlands; 6 Einthoven Laboratory for Experimental Vascular Medicine, Leiden University Medical Center, Leiden, The Netherlands; 7 Department of Human Genetics, Leiden University Medical Center, Leiden, The Netherlands; 8 Department of Internal Medicine, Division of Endocrinology, Leiden University Medical Center, Leiden, The Netherlands; 9 Center for Proteomics and Metabolomics, Leiden University Medical Center, Leiden, The Netherlands; 10 Department of Biomedical Data Sciences, Section of Molecular Epidemiology, Leiden University Medical Center, Leiden, The Netherlands; 11 Department of Epidemiology, Erasmus Medical Center, Rotterdam, The Netherlands; 12 Division of Analytical Biosciences, Leiden Academic Center for Drug Research, Leiden University and The Netherlands Metabolomics Centre, Leiden, The Netherlands; 13 Department of Epidemiology and Biostatistics, Amsterdam University Medical Center, Amsterdam, The Netherlands; 14 Julius Center for Health Sciences and Primary Care, University Medical Center Utrecht, Utrecht, The Netherlands; 15 Centre for Clinical Transfusion Research, Sanquin Research, Leiden, The Netherlands; 16 Jon J van Rood Centre for Clinical Transfusion Research, Leiden University Medical Centre, Leiden, The Netherlands; 17 TIAS, Tilburg University, Tilburg, The Netherlands; 18 Department of Clinical Epidemiology, Leiden University Medical Centre, Leiden, The Netherlands; 19 Department of Cardiology, University Medical Center Groningen, University of Groningen, Groningen, The Netherlands; 20 Center for Clinical Transfusion Research, Sanquin Research, Leiden, The Netherlands; 21 Department of Orthopedics, Leiden University Medical Centre, Leiden, The Netherlands; 22 Department of Genetics, University Medical Center Groningen, University of Groningen, Groningen, The Netherlands; 23 Department of Pediatrics, University Medical Center Groningen, University of Groningen, Groningen, The Netherlands; 24 Department of Neurology, Leiden University Medical Center, Leiden, The Netherlands; 25 Department of General Practice, The EMGO Institute for Health and Care Research, VU University Medical Center, Amsterdam, The Netherlands; 26 Department of Cardiology, Division Heart and Lungs, University Medical Center Utrecht and the Julius Center for Health Sciences and Primary Care, University Medical Center Utrecht, Utrecht, The Netherlands; 27 Thorax Centre, Erasmus Medical Centre, Rotterdam, The Netherlands; 28 Department of General Practice and Elderly Care Medicine, VU University Medical Center, Amsterdam, The Netherlands; 29 Division of Human Nutrition and Health, Wageningen University, Wageningen, The Netherlands; 30 Department of Radiology, Erasmus University Medical Center Rotterdam, Rotterdam, The Netherlands; 31 Department of Neurology, Erasmus University Medical Center Rotterdam, Rotterdam, The Netherlands; 32 Department of Rheumatology, Leiden University Medical Center, Leiden, The Netherlands; 33 Department of Internal Medicine, Division of Gerontology and Geriatrics, Leiden University Medical Centre, Leiden, The Netherlands; 34 Department of Orthopaedics, Leiden University Medical Center, Leiden, The Netherlands; 35 Department of Internal Medicine, Radboud Center for Infectious Diseases, Radboud University Medical Center, Nijmegen, Netherlands; 36 Department for Genomics & Immunoregulation, Life and Medical Sciences Institute (LIMES), University of Bonn, Bonn, Germany; 37 Department of Psychiatry, VU University Medical Center, Amsterdam, The Netherlands; 38 Department of Internal Medicine, Maastricht University Medical Center (MUMC+), Maastricht, The Netherlands; 39 School for Cardiovascular Diseases (CARIM), Maastricht University, Maastricht, The Netherlands; 40 Neurochemistry Laboratory, Clinical Chemistry Department, Amsterdam University Medical Center, Amsterdam Neuroscience, Amsterdam, The Netherlands; 41 Department of Cell and Chemical Biology, Leiden University Medical Center, Leiden, The Netherlands; 42 Department of General practice, Amsterdam University Medical Center, Amsterdam, The Netherlands; 43 Department of Critical Care, University Medical Center Groningen, Groningen, The Netherlands; 44 UMC Utrecht, Department of Rheumatology & Clinical Immunology, Utrecht, The Netherlands; 45 Department of Clinical Epidemiology, Biostatistics, and Bioinformatics, Academic Medical Centre, University of Amsterdam, Amsterdam, The Netherlands; 46 Department of Cardiology, Leiden University Medical Center, Leiden, The Netherlands; 47 Sequencing Analysis Support Core, Leiden University Medical Center, Leiden, The Netherlands; 48 Leiden Computational Biology Center, Leiden University Medical Center, Leiden, The Netherlands; 49 Department of Pattern Recognition and Bioinformatics, Delft University of Technology, Delft, The Netherlands; 50 Max Planck Institute for Biology of Ageing, Cologne, Germany; 51 Department of Psychiatry, Amsterdam UMC, University of Amsterdam, Amsterdam, The Netherlands

**Keywords:** Lipidomics, Metabolomics, Genome-wide association studies, Quantitative trait loci

Correction to: *Nature Communications* 10.1038/s41467-019-13770-6, published online 7 January 2020.

The original version of the Supplementary Information associated with this Article included an incorrect Supplementary Data 1 file, in which additional delimiters were included in the first column for a number of rows, resulting in column shifts for some of these rows. The HTML has been updated to include a corrected version of Supplementary Data 1; the original incorrect version of Supplementary Data 1 can be found as Supplementary Information associated with this Correction.

In addition, the original version of this Article contained an error in the author affiliations. An affiliation of Abdel Abdellaoui with Department of Psychiatry, Amsterdam UMC, University of Amsterdam, Amsterdam, The Netherlands was inadvertently omitted. This has now been corrected in both the PDF and HTML versions of the Article.

## Supplementary information

Supplementary Data 1

