# Supplementary material for: Heritability estimates for 361 blood metabolites across 40 genome-wide association studies
Source: Nat Commun. 2020 Jan 7;11:39. doi: 10.1038/s41467-019-13770-6 (PMC6946682; doi:10.1038/s41467-019-13770-6)
Supplement: Supplementary file 2 — Supplementary Information [file 41467_2019_13770_MOESM2_ESM.pdf]

Supplementary Information for:

Hagenbeek et al., Heritability estimates for 361 blood metabolites across 40 genome-wide association studies

Supplementary Notes

Supplementary Note 1. four-variance component models with HMDB classes

Metabolite SNPs were curated from 40 GWA and (exome-) sequencing studies (Supplementary Table 1; Supplementary Data 1) and all metabolites were categorized by HMDB super class, class and subclass (see Methods). For each class represented in our metabolite data (12) we created GRMs capturing solely the metabolite loci for metabolites of this specific class ($h^2_{Class\text{-}hits}$). Metabolite loci for each class were identified by including all published metabolite-SNP associations of the relevant class in a clumping procedure ($r^2 = 0.1$, radius = 500kb). The lead SNPs identified by the clumping procedure were then used as input to create LD-corrected GRMs in LDAK (version 4.9). We used the same technique to create corresponding non-class GRMs, which used included the lead SNPs of all metabolite-SNP associations, excluding the relevant class loci and LD proxies ($h^2_{notlass\text{-}hits}$). This resulted in 4-variance component models, as resolved in the GCTA software (version 1.91.7). Figure 1 provides an overview of the four-variance component models, including the specification of the underlying GRMs.

The 4-variance component models for the 12 different classes of metabolites had high degrees of non-convergence (37.9% total; Supplementary Table 2). Non-convergence of the 4-variance component models appears to be associated with the number of SNPs in the class-specific GRMs, with GRMs with a small number of SNPs displaying more convergence issues (Supplementary Table 2). To address the convergence issues we recoded the HMDB metabolite classes into HMDB metabolite super classes, reducing the 12 classes to 5 classes (Supplementary Table 3). Metabolites belonging to the organic nitrogen compounds, organic oxygen compounds and protein classes were not analyzed as these super classes consisted of only a single class, which had poor convergence. Combining classes into 2 super classes with a larger number of SNPs in the GRM results in a convergence rate of 97.8% (Supplementary Table 3). The complete results for the final 4-variance component models have been described in the main text of the manuscript.

Supplementary Note 2. Supplementary information metabolite profiling

Nightingale Health ^{1}H-NMR platform

Metabolic biomarkers were quantified from plasma samples using high-throughput proton nuclear magnetic resonance spectroscopy (^{1}H-NMR) metabolomics (Nightingale Health Ltd, Helsinki, Finland; formerly Brainshake Ltd.). This method provides simultaneous quantification of routine lipids, lipoprotein subclass profiling with lipid concentrations within 14 subclasses, fatty acid composition, and various low-molecular weight metabolites including amino acids, ketone bodies and glycolysis-related metabolites in molar concentration units. Details of the experimentation and epidemiological applications of the NMR metabolomics platform have been reviewed previously[1,2]. In short the Nightingale Health ^{1}HNMR platform protocol is as follows[1,2]:

Before the NMR measurements, samples are mixed with a sodium phosphate buffer (75 mmol/L Na_2HPO_4 in 80%/20% H_2O/D_2O, pH 7.4; including also 0.08% sodium 3-(trimethylsilyl)propionate-2,2,3,3-d_4 and 0.04% sodium azide) and moved to the NMR tubes. A PerkinElmer JANUS Automated Workstation with an 8-tip dispense arm with Varispan is responsible for the liquid handling. Samples are transferred to 96-well plates, with every plate includes two quality control (QC) samples (consistency of quantifications), a serum mimic and a mixture of two low-molecular–weight metabolites (performance automated liquid handler and spectrometer).

The laboratory setup combines a Bruker AVANCE III 500 MHz and Bruker AVANCE III HD 600 MHz spectrometers, both with the SampleJet robotic sample changer. The 500 MHz spectrometer is furnished with a selective inverse room temperature probe head, the 600 MHz spectrometer is furnished with a cryogenically cooled triple resonance probe head (CryoProbe Prodigy TCI). The 500 MHz and 600 MHz spectrometers can both automatically collect the lipoprotein (LIPO) and low-molecular-weight metabolites (LMWM) with standardized parameters. After spectroscopy, the samples are processed manually with a standardized lipid extraction procedure with multiple extraction steps using an Integra Biosciences VIAFLO 96 channel electronic pipette. These lipid extracts are again analyzed, in full automation, with the 600 MHz spectrometer with a standard parameter set.

Initial data processing is handled by the computers controlling the spectrometers. Processing includes automated phasing and Fourier transformation of the NMR spectra. In house-algorithms located on a centralized server process the spectral data further, which includes baseline removal, background control,

checking for missing or extra peaks, and spectral area-specific signal alignments. Regression modelling is performed to quantify the molecular data for those spectral areas that pass QC.

UPLC-MS lipidomics platform

Plasma lipid profiling was performed at the division of Analytical Biosciences at the Leiden Academic Center for Drug Research at Leiden University/Netherlands Metabolomics Centre. Study samples were first randomized and aliquotted by block. In total, the samples were divided into five blocks of seven batches each. Each of these batches included blanks, calibration lines, 9 within-batch replicates, 5 out of batch (i.e., between-batch) replicates, QC samples and approximately 83 study samples. Samples of 10µl of plasma were taken in duplicate. QC samples were added after every 10 samples. The QC sample pool was made by combining 50µl from every sample aliquotted to the first block. This pool was used as QC samples throughout the entire study. These QC samples are used to assess data quality and to correct for instrument response variability over the entire experiment. Sample preparation for positive lipids was done by isopropyl alcohol (IPA) extractions in which 1000µl of internal standards are added to the 10µl of plasma. The sample including both plasma and internal standards was centrifuged at 12 * 1000 RPM (±200 RPM) for 10 minutes at room temperature (25°C). Protein was precipitated and the lipids were extracted into the organic phase (IPA) and transferred to vials for LC-MS analysis.

The lipids were analyzed with an Ultra-High-Performance Liquid Chromatograph directly coupled to an Electrospray Ionization Quadruple Time-of-Flight high resolution mass spectrometer (UPLC-ESI-Q-TOF; Agilent 6530, San Jose, CA, USA) that uses reference mass correction. These reference mass compounds act as internal standards for the mass axis and correct for minor scan-to-scan fluctuations between runs[3]. For liquid chromatographic separation a ACQUITY UPLC HSS T3 column (1.8µm, 2.1 ∗ 100mm) was used with a flow of 0.4 ml/min over a 16 minute gradient. Lipid detection was done using a full scan in the positive ion mode. The raw MS data were pre-processed using Agilent MassHunter Quantitative Analysis software (Agilent, Version B.04.00).

To compensate for any variations in the LC-MS analysis method the following three steps were carried out: 1) blank samples were used to subtract background contributions from the samples; 2) the lipid response was calculated as the peak area ratios of the target lipid to the respective internal standard; and 3) in-house developed algorithms were used to compensate for shifts in the sensitivity of the mass spectrometer over the different batches, using the pooled QC samples. Finally, all peaks were checked manually.

Leiden ^{1}H-NMR platform (for small metabolites)

Metabolomic profiling on this platform was performed at the Center for Proteomics and Metabolomics, Leiden University Medical Center, Leiden, the Netherlands. Before measurement, the containers containing the thawed serum samples (4°C) were inverted 10 times, thereby mixing the samples. Using a Gilson 215 liquid handler with a Bruker SampleTrack system the samples (300 µL) were mixed with 300 µL 75 mM disodium phosphate buffer in H_2O/D_2O (80/20) with a pH of 7.4 comprising 6.15 mM NaN3 and 4.64 mM sodium 3-[trimethylsilyl] d4-propionate (TSP). A modified Gilson 215 tube filling station were used to transfer samples to 5 mm SampleJet NMR tubes in 96 tube racks. While queued for acquisition the samples were kept at 6°C on a SampleJet sample changer[4].

The Leiden ^{1}H-NMR spectroscopy experiment of EDTA-plasma samples used a 600 MHz Bruker Advance II spectrometer (Bruker BioSpin, Karlsruhe, Germany) using the 2D J-resolved (JRES) and CPMG (Carr-Purcell-Meiboom-Gill) methods. Low quality spectra were excluded in the QC procedure before the intensities were obtained by linear models from the CPMG spectra. The linear model was created by relating the well-resolved peaks in the JRES spectrum to the intensity profile from the CPMG spectrum. Using the Simplex method the JRES spectra were fitted to Gauss-Lorents line-shapes. This platform could annotate 42 metabolites by a combination of chemical shift interpretation and by interpreting the cross-correlation between the peaks and samples spiked with pure compounds[4].

Biocrates Absolute-IDQ^{TM} p150 platform

The Biocrates Absolute-IDQ^{TM} p150 (Biocrates Life Sciences AG, Innsbruck, Austria) metabolomics platform on serum samples was analysed at the Metabolomics Facility of the Genome Analysis Centre at the Helmholtz Centre in Munich, Germany as specified by the manufacturer. The Biocrates Absolute-IDQ^{TM} p150 kit confirms to FDA-Guidelines, and is described by the manufacturer in manual UM-P150. Briefly, the sample preparation involves pipetting the serum samples to 96-well plates which includes internal standards. The samples are then dried under nitrogen stream and the amino acids are derivatized with 5% phenylisothiocyanate reagent. After drying of the samples the metabolites and internal standards are extracted with 5mM of ammonium acetate in methanol, centrifuged through a filter membrane and diluted into the MS running solvent[5]. The Biocrates Absolute-IDQ^{TM} p150 platform uses flow injection analysis coupled to tandem mass spectrometry (MS/MS) and metabolites and internal standards are quantified by selected reaction monitoring (SMR)[6]. Quality assessment and quantification of metabolite concentrations have been performed with MetIQ^{TM} as included with the metabolomics kit.

Supplementary Note 3. Comparison of weighted vs. unweighted GRMs and clumped vs. not-clumped GRMs

Simulations of using a weighted vs. unweighted GRMs

Genome-wide complex trait analysis (GCTA)[7] was used to simulate 20 traits with heritabilities of 20%, 40%, 60% and 80% under the assumption that 1000 random single nucleotide polymorphisms (SNPs; (e.g., polygenic traits) or 5 random SNPs (e.g., near monogenic traits) are causally underlying these traits. Data underlying these simulations were the cross-imputed genotype sets (see Methods). SNP heritability (h^2_{SNP}) analyses were run in GCTA for all simulated traits using a weighted genetic relatedness matrix (GRM; LDAK[8,9]) and an unweighted GRM in order to compare the accuracy of the h^2_{SNP} estimates. The simulations show that estimated heritabilities from both GRMs deviate slightly from the simulated heritability.

Under the polygenic assumption, the weighted GRM shows an upwards bias for lower simulated heritabilities (Supplementary Figure 4a), while the unweighted GRM shows a larger downwards bias for higher simulated heritabilities (Supplementary Figure 4b). Under near monogenic assumptions the differences between the weighted and unweighted GRMs largely disappear (Supplementary Figure 4c-4d). The accuracy for both GRMs under near monogenic assumptions appeared to be quite similar, therefore, additional simulations under near monogenic assumptions were run as previous studies have shown that relatively large proportions of variance in serum metabolite levels can be explained by a relatively small number of genetic variants[10–13]. Three sets of 5 'causal' SNPs were selected, with one set of SNPs in low linkage disequilibrium (LD) with each other, a set of medium LD SNPs and a set of SNPs in high LD. High, medium and low LD was assigned based on the LD-weighting as calculated by LDAK. For each of these SNP sets 20 traits with heritabilities of 10-50% were simulated and h^2_{SNP} estimates were compared for the weighted and unweighted GRMs. Across all LD sets the unweighted GRM underestimated the simulated heritability, weighted GRMs appeared to have smaller biases (Supplementary Figure 5). However, as genotyped or imputed datasets as used here already includes the LD-structure in the data and likely biases the simulations in favour of weighted GRMs, we are only able to conclude that, as our simulations are likely biased to favor LDAK, LDAK does at least as well as GCTA.

Clumped vs. not-clumped weighed and unweighted GRMs

After curating all metabolite-SNP associations of the past decade we have identified 35,138 unique metabolites SNPs (see Methods and Supplementary Figure 1). Many of these SNPs are from the same genetic loci and likely describe the same association for different metabolites. Including all SNPs may

induce upward bias of the heritability estimates. Therefore, in constructing the weighted GRMs from the class-specific and not-class-specific metabolite loci we first clump all metabolite-SNP associations (Supplementary Figure 1) to obtain independent metabolite loci. However, clumping of SNPs before creating weighted GRMs may potentially remove real signal and result in underestimation of the heritability estimates. To investigate if clumped indeed results in downward bias of our heritability estimates we compared clumped and not-clumped LDAK metabolite GRMs. We used the GCTA power calculator to compare the estimated SE's and power for the different GRMs included in our manuscript using clumped and not-clumped SNPs (https://cnsgenomics.shinyapps.io/gctaPower/).

The differences between the GRMs using clumped or not-clumped SNPs are very small (Supplementary Data 12). Across all 5 GRMs the average mean differences between the clumped and not-clumped GRMs are negative, indicating that clumped GRMs results in smaller SE's, though with a minimal difference (Supplementary Table 16). Similarly, the average power differences are zero or positive (again with minimal differences), indicating that clumped GRMs are as powerful or more powerful than not-clumped GRMs (Supplementary Data 12). While the gain of using a clumped weighted LDAK GRM versus a not-clumped LDAK GRM is small, we feel that the given the large SE's we've observed in our study, the choice for clumped weighted LDAK GRMs is valid. However, by clumping the SNPs prior to creating the weighed GRM the differences between weighted and unweighted GRMs become very small (Supplementary Data 13). A final worry about the use of a weighted GRM is the effect of thresholding[14] in order to obtain the GM including only the closely related individuals. As can be seen in the scatterplot (Supplementary Figure 6) of the weighted (LDAK) vs unweighted (GCTA) thresholded GRM, there are some discrepancies in the individuals identified as not closely-related (set to zero) in one or the other methods (horizontal/vertical outliers bottom left corner graph). However, the number of relationships not classified as unrelated by both methods is very low. Fourteen pairs were classified as unrelated by the LDAK method, but not by GCTA, and two pairs were classified as unrelated by the GCTA method, but not by LDAK. Therefore, the effect of using LDAK or GCTA to create the GRM retaining only the relationships among closely related individuals will be minimal.

Supplementary Note 4. Covariate determination

Generalized estimation equation models

The contribution of covariates to the metabolite levels after quality control was estimated using generalized estimation equation models (GEE). GEE models were fitted in the R programming language (R version 3.5.1) using the GEE-package (version 4.13-19) with the Gaussian link function for continuous data and the 'exchangeable' option to correct for the correlation structure due to family resemblance with 100 iterations. All GEE models were done on a subset of the data in which individuals with missing data on the covariates were excluded. Matrix Spectral Decomposition (MSD)[15] was applied to estimate the number of independent variables in the correlation matrix of all metabolites that passed quality control (QC). Correction for multiple testing was done by a Bonferroni correction for the number of independent variables (α= 0.05/ N independent variables), as in van Dongen et al. (2015)[16]. MSD identified 93 independent variables, therefore, the significance threshold for the gee analyses was $p \leq 0.0005$.

Covariates included sex, age at blood draw, body mass index (BMI), smoking status (never, former, current), use of sex-hormones (y/n, defined as medications belonging to the 'G03 – sex hormones and modulators of the genital system' ATC class), use of systemic hormones (y/n, here systemic hormone use is defined as including medication belong to the following ATC classes: 'H01 – pituitary and hypothalamic hormones and analogues', 'H02 – corticosteroids for systemic use', 'H03 – thyroid therapy', and 'H04 – pancreatic hormones'), use of medication for diabetes ('A10 – drugs used in diabetes), use of medication for hypertension (y/n, defined as medications belong to the following ATC classes: 'C02 – antihypertensives'. 'C03 – diuretics', 'C04 – peripheral vasodilators', 'C05 – vasoprotectives', 'C07 – beta blocking agents', 'C08 – calcium channel blocker', and 'C09 – agents acting on the renin-angiotensin system'), use of medication for chronic obstructive pulmonary disease (COPD; y/n, defined as medications belong to the ATCH classes 'R01 – nasal preparations' and 'R03 – drugs for obstructive airway diseases), and use of anti-inflammatory medications (y/n, defined as medications belonging to the following ATC classes: 'L01 – antineoplastic agents', 'L02 – endocrine therapy', 'L03 – immunostimulants', 'L04 – immunosuppressants', 'M01 – anti-inflammatory and antirheumatic products' and 'M02 – topical products for joint and muscular pain'). We also considered the following technical covariates: genotyping chip (AXIOM, AFFY6, ILL660, ILL1M, ILLGSA, PERAFF or GONL sequencing), measurement batch (not included for the UPLC-MS Lipidomics platform) and population stratification by including the first 10 genetic principal components (PC) as based on the Dutch population[17].

Metabolite levels showed associations with sex, age, BMI, smoking status and use of sex-hormones. After correction for multiple testing 79.9% of the metabolites had a significant association with age (295/369), 78.6% with sex (290/369), 50.4% with use of sex-hormones (186/369), 48% with BMI (177/369) and 32.8% with smoking status (121/369; Supplementary Data 14). For the significantly associated metabolites, we observed overall higher metabolite levels for older individuals, individuals with a higher BMI and individuals using sex-hormones (Supplementary Data 14). Overall, lower metabolite levels were observed for current smokers as compared with former or never smokers, while the effect of sex on metabolite levels varied greatly (Supplementary Data 14). Only few metabolites were associated with diabetes medication (0.8%), anti-hypertension medication (3.3%) or the use of systemic hormones (1.6%; Supplementary Data 14). COPD medication and anti-inflammatory medication were not significantly associated with metabolite levels of any of the four platforms after correction for multiple testing (Supplementary Data 14).

Comparing three different two-variance component models in GCTA

In order to determine the influence of covariates on the heritability estimates we ran three different models. Aschard et al. (2015) showed that including a heritable trait as covariate in GWAS analysis, or when calculating heritability for a trait, might lead to biased estimates for the trait of interest[18]. For this reason we compared the full and reduced two-variance component models, both included (potentially) heritable covariates, with a sparse two-variance component model, that did not include any (potentially) heritable covariates. The full model included all covariates as were included in the GEE models, the reduced model excluded COPD medication use, anti-inflammatory medication use and use of systemic hormones, as these were overall not significantly associated with metabolite levels across the different platforms, and finally the sparse model included only sex and age at blood draw. In addition all models also included genetic PCs, genotyping chip and when applicable measurement batch (see Supplementary Table 5).

The total heritability (h^2_{total}) and h^2_{SNP} estimates for all metabolites across all models can be found in Supplementary Data 15. The mean and median h^2_{total} and h^2_{SNP} estimates across all metabolites per model are provided in Supplementary Table 6. Aschard et al. (2015) predicted that models including heritable covariates will have increased heritability estimates as compared with the models without heritable covariates. In contrast to this prediction, we observed slightly higher mean and median h^2_{total} and h^2_{SNP} estimates when considering the sparse model as compared with the full and/or reduced model. These

results seem to imply that the inclusion of (potentially) heritable traits generally does not bias the estimates in an upwards fashion for the metabolomics platforms under consideration.

The Log Likelihood (LogL) for each of the models for all metabolites across all four platforms have been extracted to calculate the likelihood ratio test (LRT) to compare the full model with the reduced and sparse models (Supplementary Data 15). The LRT shows that on average (99.2%) the reduced model is not a better fit for the data as compared to the full model, but the sparse model generally fits the data better (56.9%; Supplementary Data 15). Therefore, we decided to use the most sparse model in the further analyses.

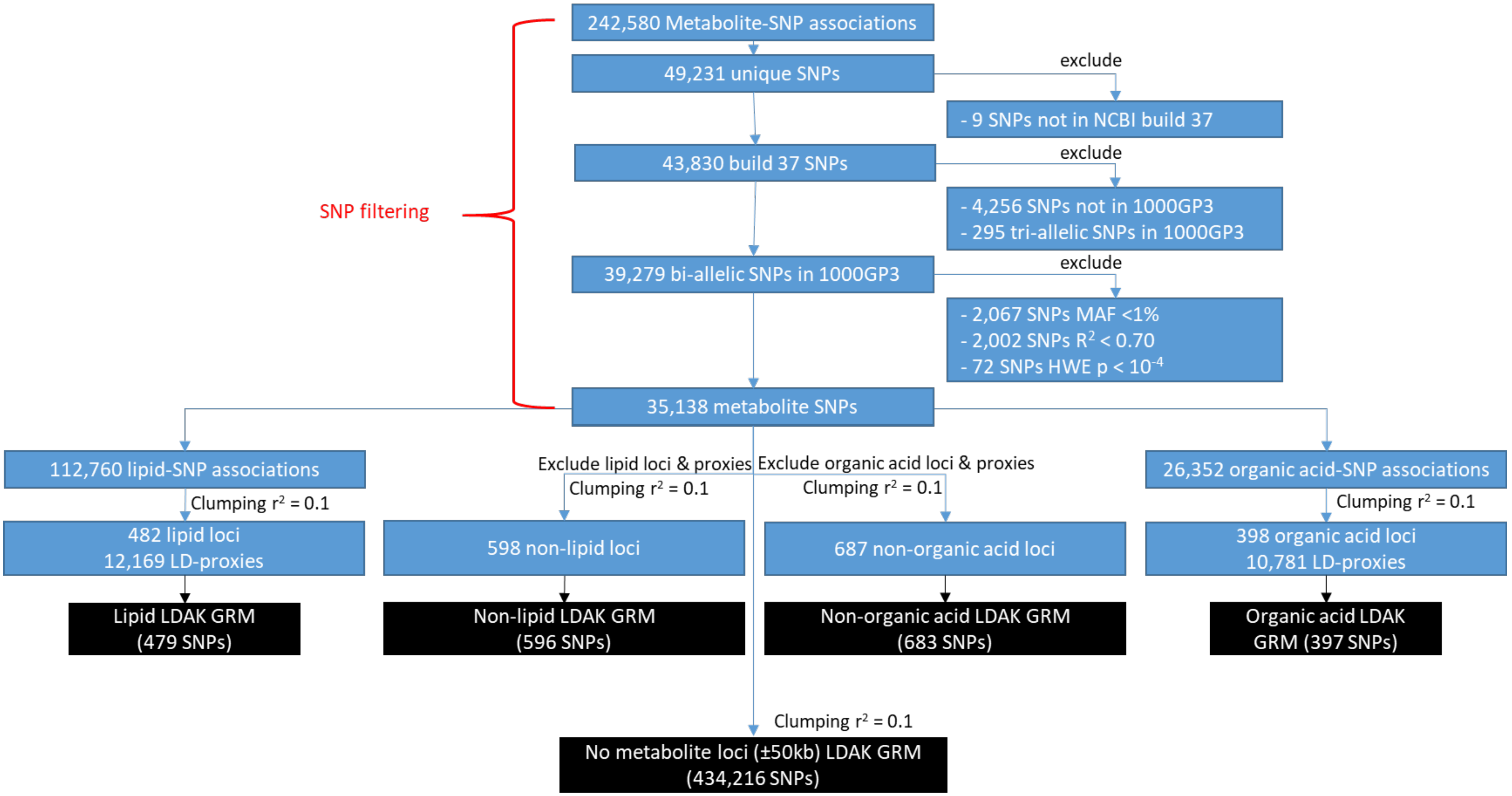
242,580 Metabolite-SNP associations
49,231 unique SNPs
exclude
- 9 SNPs not in NCBI build 37
43,830 build 37 SNPs
exclude
- 4,256 SNPs not in 1000GP3
- 295 tri-allelic SNPs in 1000GP3
SNP filtering
39,279 bi-allelic SNPs in 1000GP3
exclude
- 2,067 SNPs MAF <1%
- 2,002 SNPs R2 < 0.70
- 72 SNPs HWE p < 10-4
35,138 metabolite SNPs
112,760 lipid-SNP associations
Clumping r2 = 0.1
482 lipid loci
12,169 LD-proxies
Lipid LDAK GRM
(479 SNPs)
Exclude lipid loci & proxies
Clumping r2 = 0.1
598 non-lipid loci
Non-lipid LDAK GRM
(596 SNPs)
Exclude organic acid loci & proxies
Clumping r2 = 0.1
687 non-organic acid loci
Non-organic acid LDAK GRM
(683 SNPs)
26,352 organic acid-SNP associations
Clumping r2 = 0.1
398 organic acid loci
10,781 LD-proxies
Organic acid LDAK
GRM (397 SNPs)
Clumping r2 = 0.1
No metabolite loci (±50kb) LDAK GRM
(434,216 SNPs)

Supplementary Figure 1. Flowchart describing the filtering of metabolite SNPs and GRM construction for the 4-variance component models.

This flowchart describes how the 242,580 metabolite-SNP associations as identified from GWA and rare-variant analyses (Supplementary Table 1; Supplementary Data 1) were converted to NCBI build 37, extracted for NTR participants from the 1000GP3 imputed data and filtered on MAF, HWE and R^2 (blue boxes at top of the figure indicated by the red curly bracket). The metabolite-SNP associations of the filtered SNPs were clumped (r^2 = 0.10) to obtain the metabolite loci and LD-proxies of the lipid and the organic acids, respectively (blue). To obtain the non-superclass loci, the superclass-specific loci and LD-proxies were removed from the overall list of metabolite-SNP associations and prior to clumping (blue). The lipid-loci, not-lipid loci, organic acid loci and not-organic acid loci give rise to four GRMs, respectively, as indicated by the black boxes and arrows in the flowchart. The two additional GRMs included in the 4-variance component GREML models are based on the cross-platform imputed SNPs (see Methods), where the lipid and organic acid loci, LD-proxies and 50 kb surrounding these SNPs have been removed from one of the cross-platform GRMs (black box in flowchart).

(a)

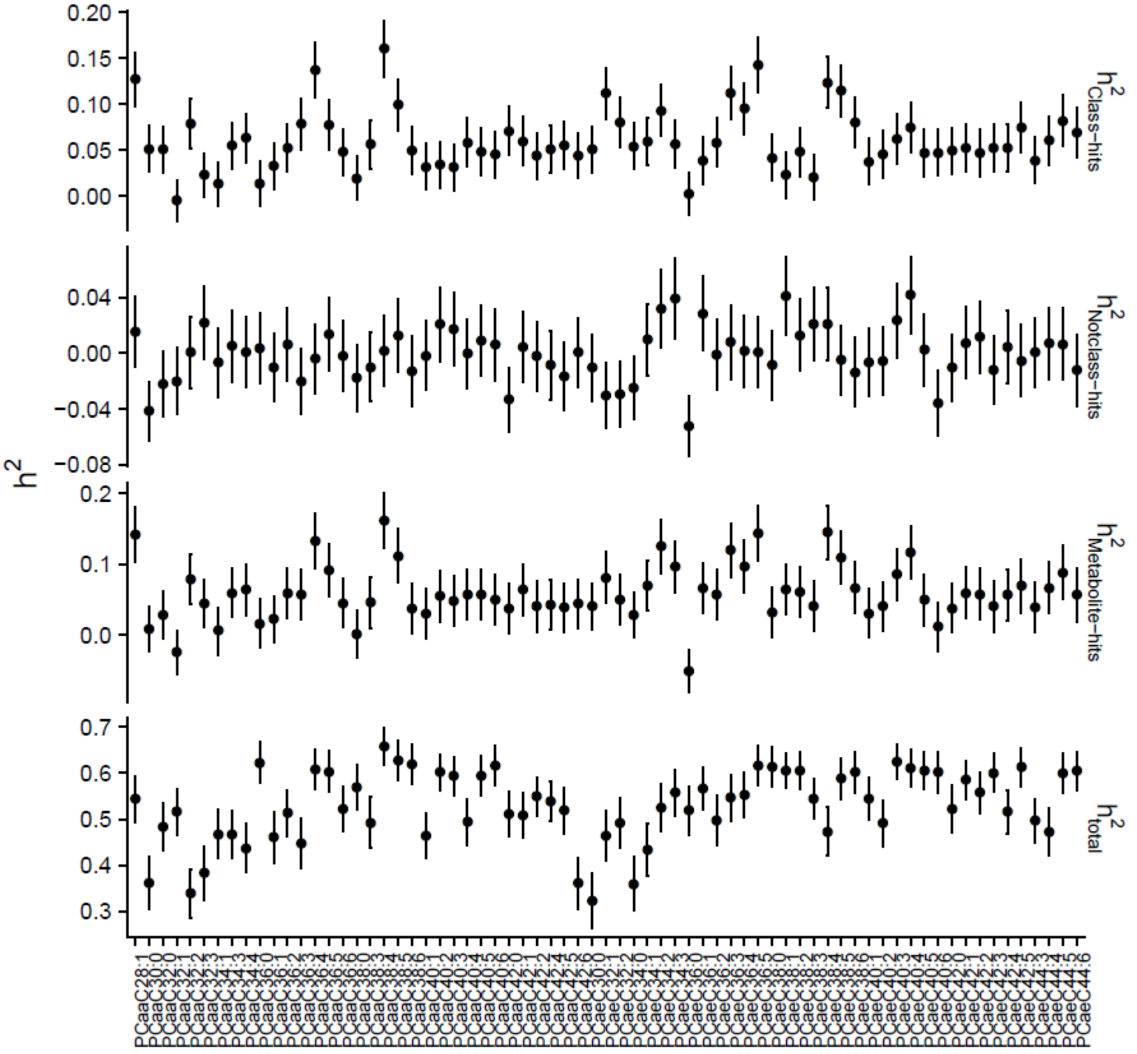
h2
0.20
0.15
0.10
0.05
0.00
h2 Class−hits
0.04
0.00
−0.04
−0.08
h2 Notclass−hits
0.2
0.1
0.0
h2 Metabolite−hits
0.7
0.6
0.5
0.4
0.3
h2 total

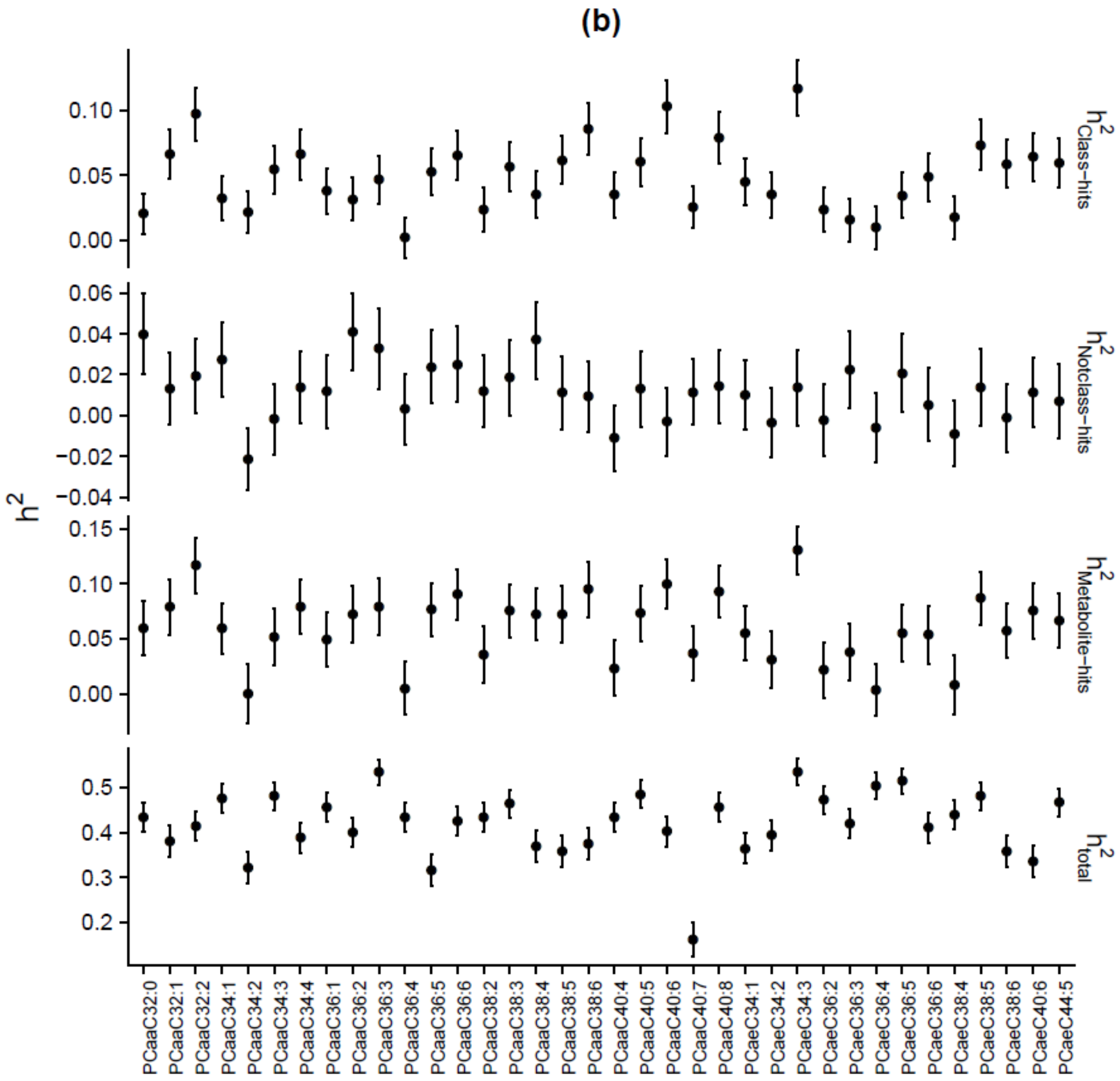
(b)
h2
h2 Class−hits
h2 Notclass−hits
h2 Metabolite−hits
h2 total
PCaaC32:0
PCaaC32:1
PCaaC32:2
PCaaC34:1
PCaaC34:2
PCaaC34:3
PCaaC34:4
PCaaC36:1
PCaaC36:2
PCaaC36:3
PCaaC36:4
PCaaC36:5
PCaaC36:6
PCaaC38:2
PCaaC38:3
PCaaC38:4
PCaaC38:5
PCaaC38:6
PCaaC40:4
PCaaC40:5
PCaaC40:6
PCaaC40:7
PCaaC40:8
PCaeC34:1
PCaeC34:2
PCaeC34:3
PCaeC36:2
PCaeC36:3
PCaeC36:4
PCaeC36:5
PCaeC36:6
PCaeC38:4
PCaeC38:5
PCaeC38:6
PCaeC40:6
PCaeC44:5

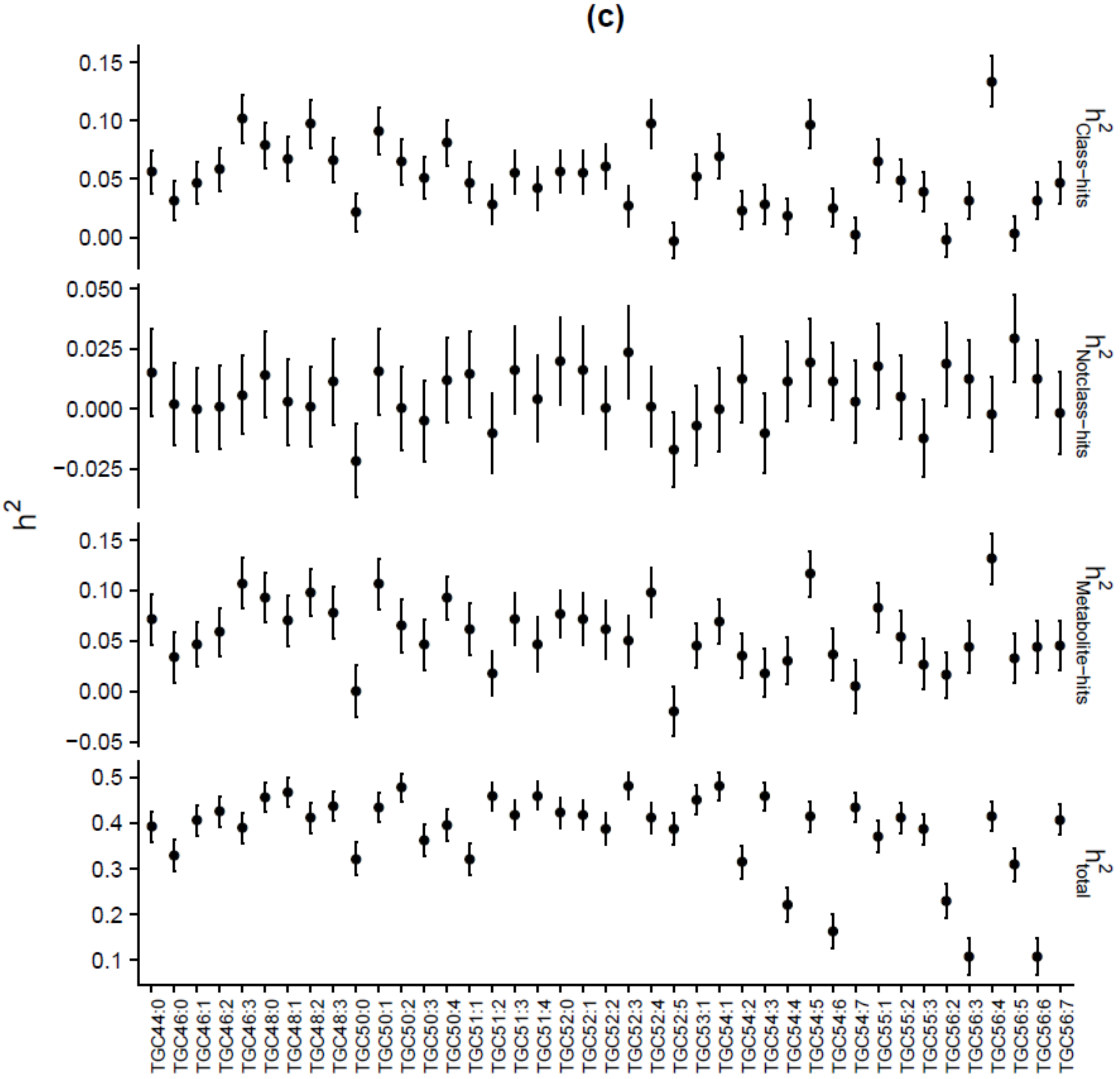

Supplementary Figure 2. Heritability estimates of h^2_{total}, $h^2_{Class\text{-}hits}$, $h^2_{Notclass\text{-}hits}$ and $h^2_{Metabolite\text{-}hits}$ with standard errors (SE) for the phosphatidylcholines (PCs) and triglycerides (TGs) ordered by the number of carbon atoms and double bonds in each species. (a) PCs as measured on the Biocrates platform. (b) PCs as measured on the UPLC-MS lipidomics platform. (c) TGS as measured on the UPLC-MS lipidomics platform. The error bars denote one SE around the point estimate. Supplementary Data 3 provides the estimates for each of the individual metabolites.

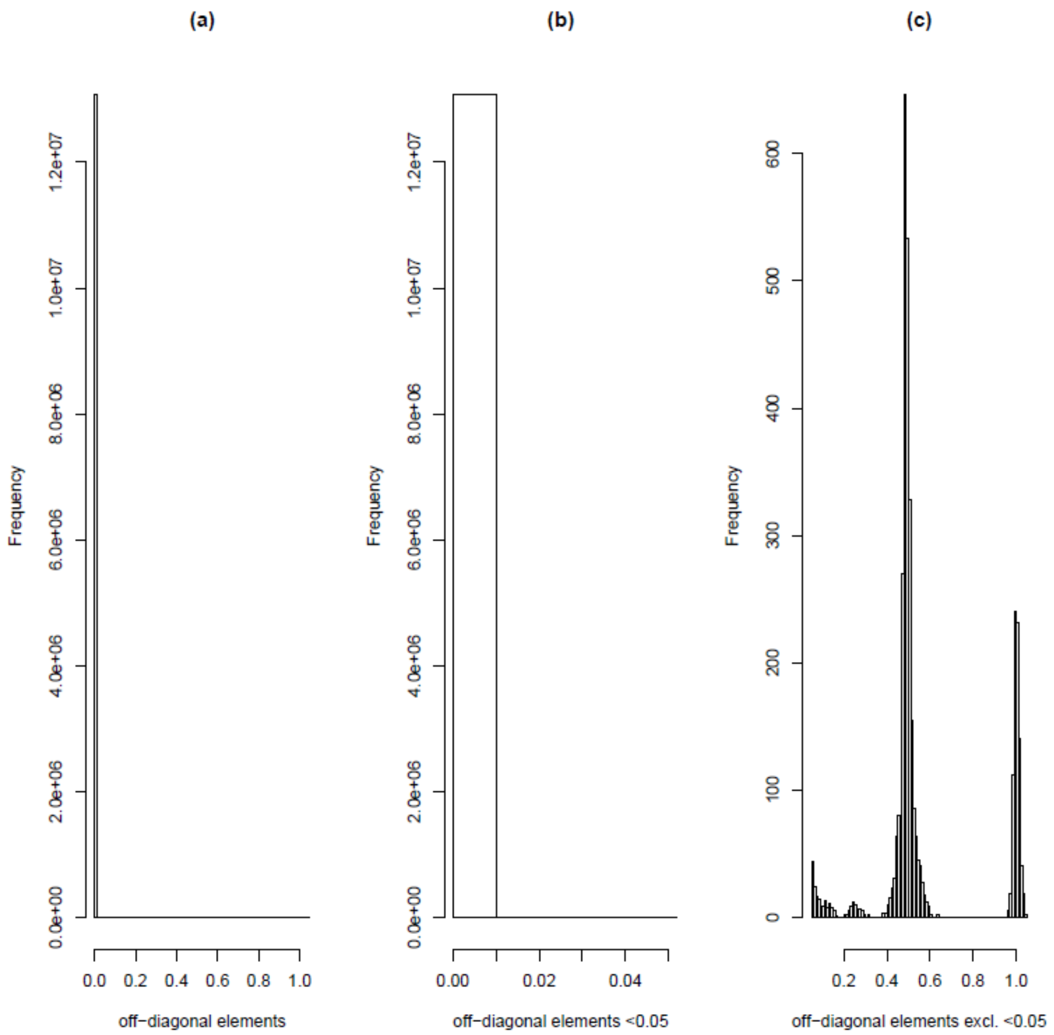

Supplementary Figure 3. Histogram of the off-diagonal elements of the Genetic Relatedness Matrix (GRM) of all participants. The off-diagonal elements represent the pairwise relatedness for all participants. (a) Histogram of all off-diagonal elements of the LDAK GRM for all participants. As a large proportion of relationships among the participants is unrelated (<0.05) this histogram is highly skewed to the left, making the relationships among related individuals undistinguishable. (b) Histogram of the off-diagonal elements in the LDAK GRM for the unrelated (<0.05) participants. (c) Histogram of all off-diagonal elements of the LDAK GRM for all related (>0.05) participants.

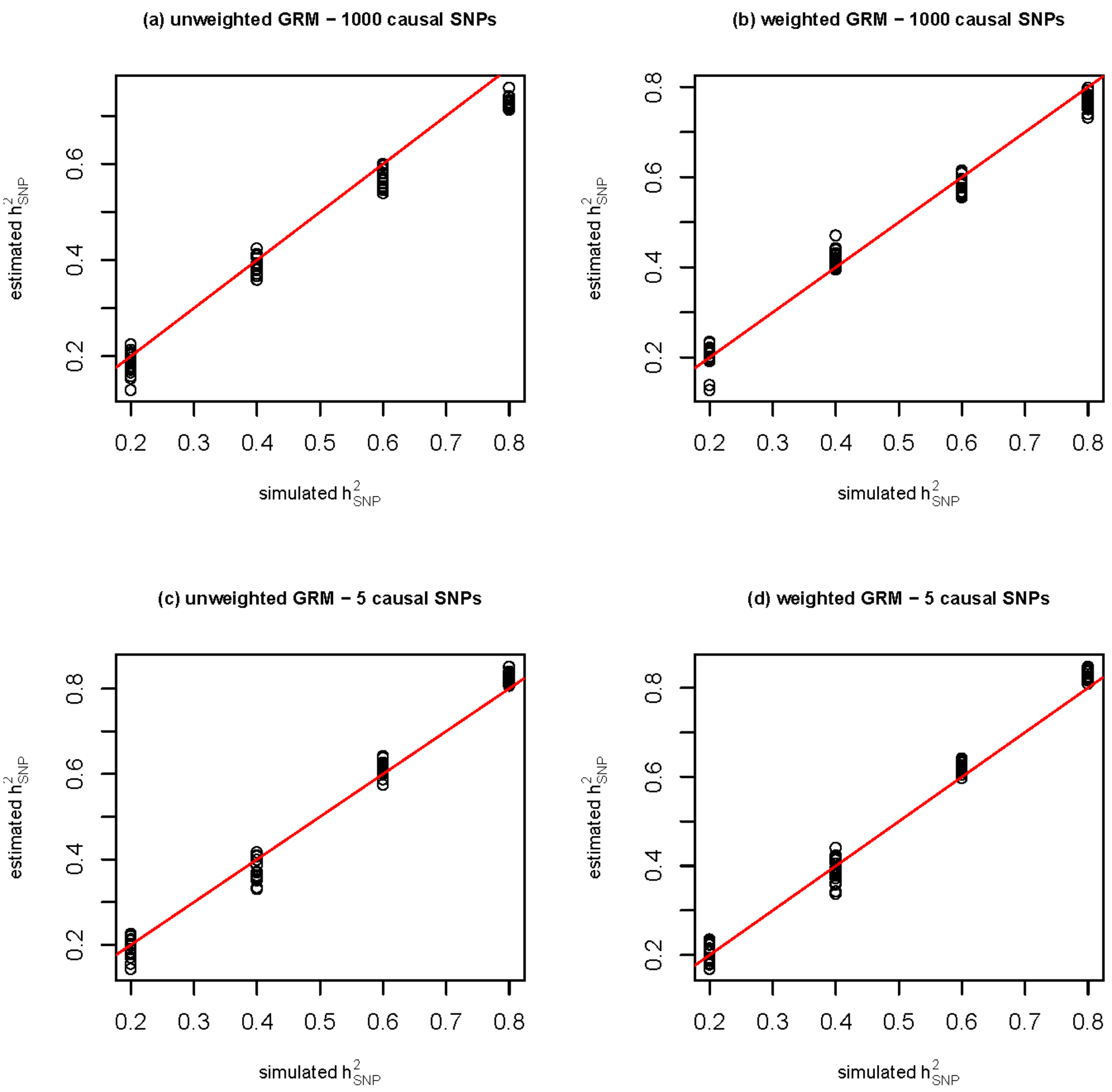

Supplementary Figure 4. GCTA results with weighted versus unweighted GRMs of 20 simulated traits under multiple heritabilities with 1000 causal SNPs or 5 causal SNPs. (a) GCTA results with unweighted GRM of 20 simulated traits under multiple heritabilities with 1000 causal SNPs. (b) GCTA results with weighted GRM of 20 simulated traits under multiple heritabilityies with 1000 causal SNPs. (c) GCTA results with unweighted GRM of 20 simulated traits under multiple heritabilities with 5 causal SNPs. (d) GCTA results with weighted GRM of 20 simulated traits under multiple heritabilities with 5 causal SNPS. The red line indicates a perfect overlap between the simulated and estimated heritabilities.

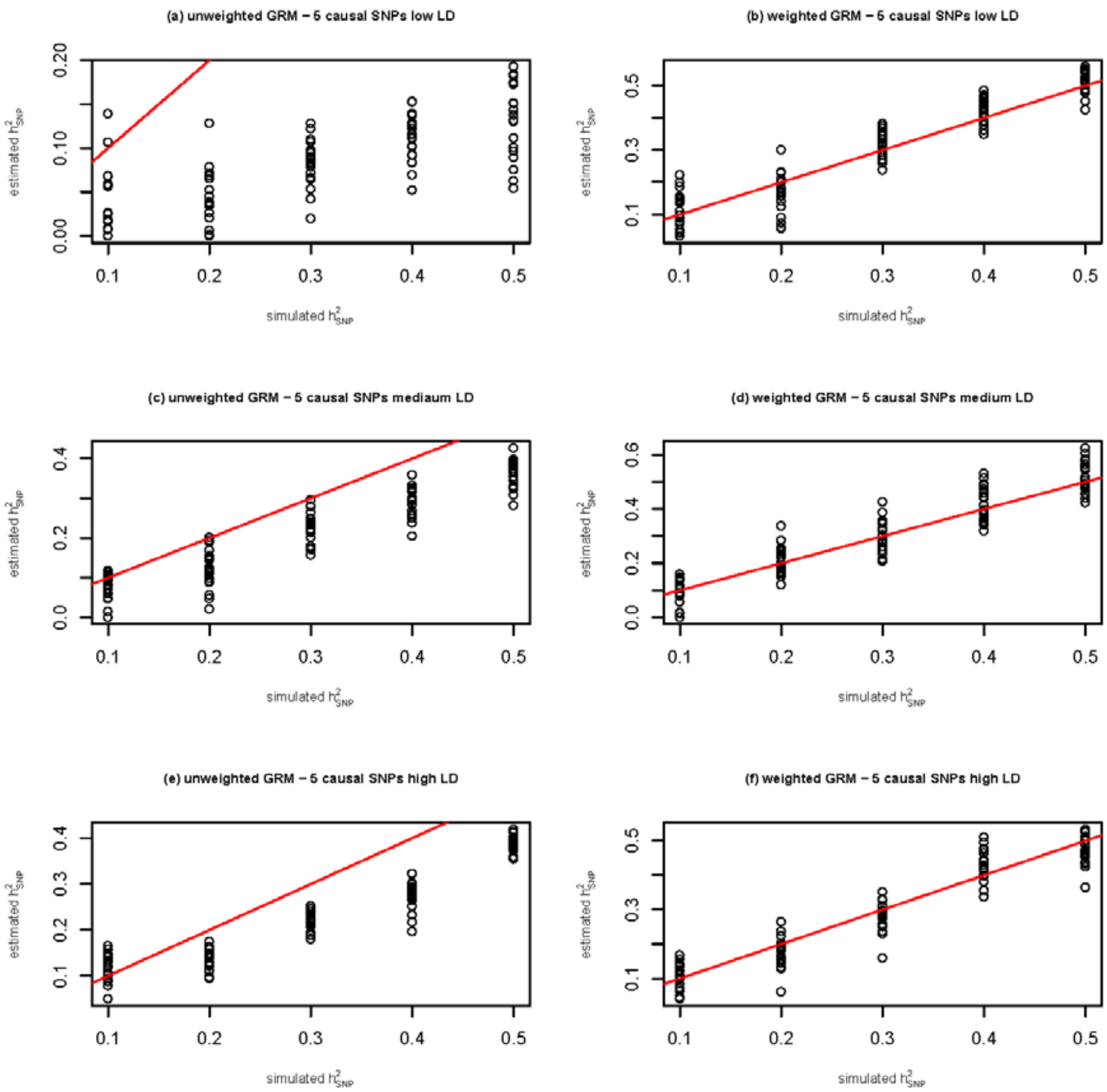

Supplementary Figure 5. GCTA results with weighted versus unweighted GRMs of 20 simulated traits under multiple heritabilities with 5 causal SNPs selected to be in low, medium or high LD. (a) GCTA results with unweighted GRMs of 20 simulated traits under multiple heritabilities with 5 causal SNPs selected to be in low LD. (b) GCTA results with weighted GRMs of 20 simulated traits under multiple heritabilities with 5 causal SNPs selected to be in low LD. (c) GCTA results with unweighted GRMs of 20 simulated traits under multiple heritabilities with 5 causal SNPs selected to be in medium LD. (d) GCTA results with weighted GRMs of 20 simulated traits under multiple heritabilities with 5 causal SNPs selected to be in medium LD. (e) GCTA results with unweighted GRMs of 20 simulated traits under multiple heritabilities with 5 causal SNPs selected to be in high LD. (f) GCTA results with weighted GRMs of 20 simulated traits under multiple heritabilities with 5 causal SNPs selected to be in high LD. The red line indicates a perfect overlap between the simulated and estimated heritabilities.

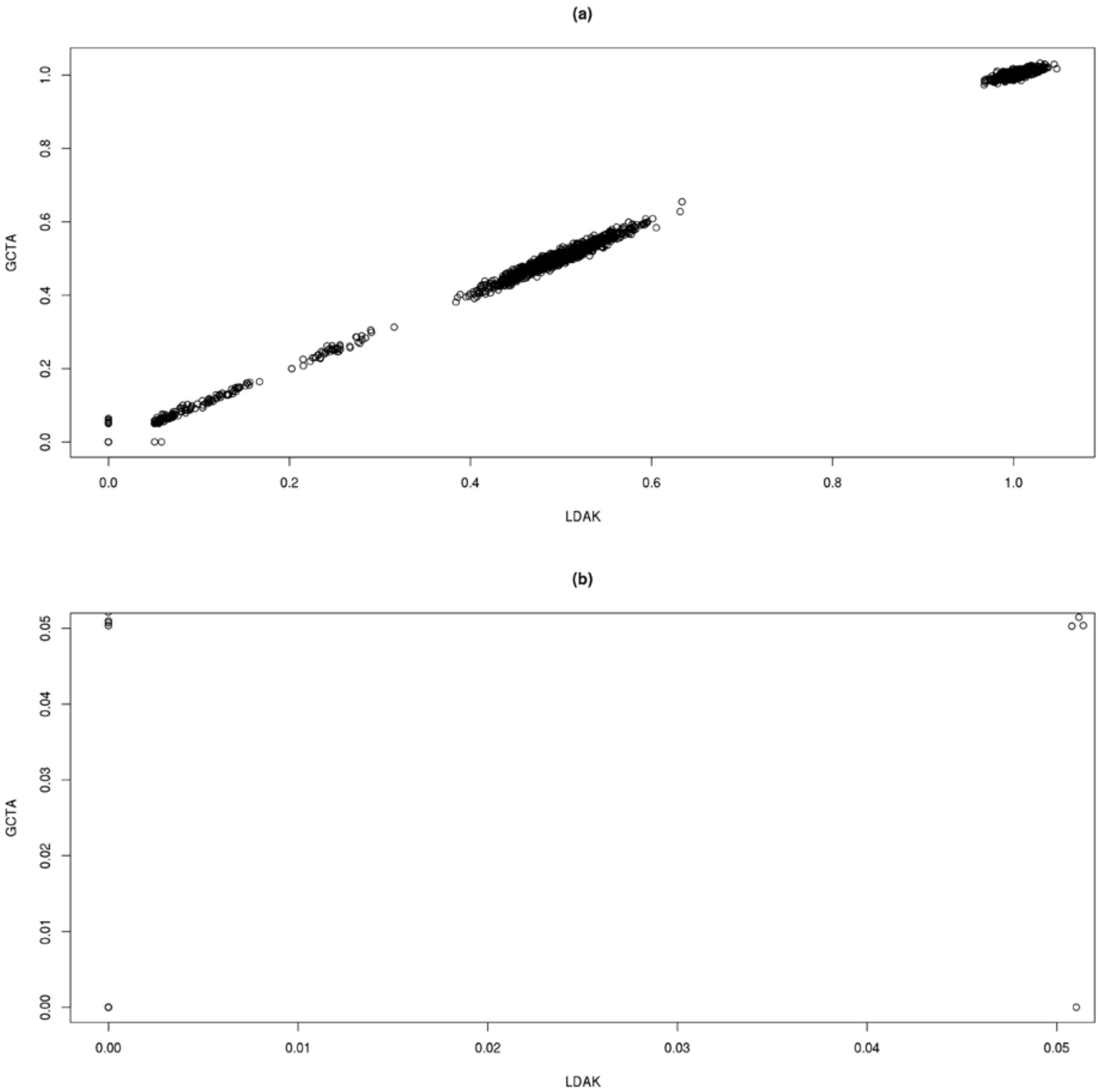

Supplementary Figure 6. Scatterplot of the off-diagonal elements of the clumped LDAK versus clumped GCTA GRMs with the relationships for unrelated individuals (<0.05) set to zero. (a) Scatterplot of all off-diagonal elements of the clumped LDAK versus clumped GCTA GRMs with the relationships for unrelated individuals (<0.05) set to zero. (b) . Scatterplot of the <0.05 off-diagonal elements of the clumped LDAK versus clumped GCTA GRMs with the relationships for unrelated individuals (<0.05) set to zero.

Supplementary Tables

Supplementary Table 1. Overview of GWA and (exome-) sequencing studies of blood metabolites published between November 2008 and October 2018.

We updated and extended the existing review for QTL discovery[19] for metabolites in European cohorts and supply the complete list of references in the Supplementary References section.

Study	*Biofluid*	*Sample (N)*	*Population*	*Study type*	*Genome build*	*Metabolomics platform(s)*	*Metabolites (N)*
(Gieger et al. 2008)[10]	Serum	284	European	GWA	NCBI build 35	Biocrates (MS)	363 + ratios
(Tanaka et al. 2009)[20]	Plasma	1,210 + 1,076	European	GWA	NCBI build 35	gas chromatography	6
(Hicks et al. 2009)[21]	Plasma & serum	4,400	European	GWA	NCBI build 36	Lipidomics (MS)	33 + 43 ratios
(Illig et al. 2010)[11]	Serum	1,809+422	European	GWA	NCBI build 36	Biocrates (MS)	163 + 26,406 ratios
(Lemaitre et al. 2011)[22]	Plasma	8,866	European	GWA	NCBI build 35	gas chromatography	4
(Suhre, Shin, et al. 2011)[23]	Serum	1,768 + 1,052	European	GWA	NCBI build 36	Metabolon (MS)	276 + 37,179 ratios
(Nicholson et al. 2011)[24]	Urine & plasma	211	European	GWA	NCBI build 37	NMR in urine & Biocrates (MS) in plasma	512 urine + 163 + ratios plasma
(Kettunen et al. 2012)[12]	Serum	8,330	European	GWA	NCBI build 36	Nightingale Health (NMR)	117 + 99 ratios
(Demirkan et al. 2012)[25]	Plasma	4,043	European	GWA	NCBI build 36	Lipidomics (MS)	153
(Tukiainen et al. 2012)[26]	Serum	8,330	European	refinement known loci	NCBI build 36	Nightingale Health (NMR)	117 + 99 ratios

Study	Biofluid	Sample (N)	Population	Study type	Genome build	Metabolomics platform(s)	Metabolites (N)
(Krumsiek et al. 2012)[27]	Serum	1,768	European	GWA	NCBI build 36	Metabolon (MS)	517
(Wu et al. 2013)[28]	Plasma	8,961	European	GWA	NCBI build 36	gas chromatography	4
(Raffler et al. 2013)[29]	Plasma	1,757	European	GWA	NCBI build 36	NMR + Biocrates + Metabolon (MS)	8,600 + 124,750 ratios
(Hong et al. 2013)[30]	Serum	402 + 489	European	GWA	NCBI build 36	MS	6,138
(Xie et al. 2013)[31]	Plasma	1,004 + 341	European	GWA	NCBI build 36	Metabolon (MS)	14
(Rhee et al. 2013)[32]	Plasma	2,076	European	GWA	NCBI build 36	MS	217
(Shin et al. 2014)[33]	Serum	7,824	European	GWA	NCBI build 36	Metabolon (MS)	486 + 98,346 ratios
(Guan et al. 2014)[34]	Plasma	8,631	European	GWA	NCBI build 36	gas chromatography	5
(Mozaffarian et al. 2015)[35]	Red blood cells + plasma	8,013	European + African American + Chinese + Hispanic	GWA	NCBI build 36	gas chromatography	5
(Lemaitre et al. 2015)[36]	Plasma	10,129	European	GWA	NCBI build 36	gas chromatography	3
(Demirkan et al. 2015)[4]	Serum	2,118	European	GWA + exome sequencing	NCBI build 36 (GWA) and 37 (exome)	'Leiden' (NMR)	42
(Yu et al. 2015)[37]	Serum	1,152 + 718 + 753	African American + European	exome sequencing + exome chip	NCBI build 37	Metabolon (MS)	1
(Draisma et al. 2015)[13]	Serum	7,478 + 1,182	European	GWA	NCBI build 36	Biocrates (MS)	129
(Tintle et al. 2015)[38]	Red blood cells	2,633	European	GWA	NCBI build 36	gas chromatography	14

Study	Biofluid	Sample (N)	Population	Study type	Genome build	Metabolomics platform(s)	Metabolites (N)
(Hu et al. 2016)[39]	Red blood cells + plasma	2,685 + 656 + 8,866 + 8,962	Chinese + European	GWA	NCBI build 36	gas chromatography	9
(Hartiala et al. 2016)[40]	Plasma	1,985 + 1,895 + 400	European	GWA	NCBI build 36	MS	9
(Kettunen et al. 2016)[41]	Serum & plasma	24,925	European	GWA	NCBI build 37	Nightingale Health (NMR) + 'Leiden' (NMR)	123
(Yet et al. 2016)[42]	Serum	1,001	European	GWA	NCBI build 36	Biocrates & Metabolon (MS)	648
(Yazdani et al. 2016)[43]	Serum	1,456	European	whole genome sequencing	NCBI build 37	Metabolon (MS)	16
(Fall et al. 2016)[44]	Plasma	1,138 + 970 + 1,630	European	GWA + look-up	only rsids used	non-targeted MS	15
(Rhee et al. 2016)[45]	Plasma	2,076 + 1,528	European	exome chip	NCBI build 37	Metabolon (MS) + other MS	217
(Yu et al. 2016)[46]	Serum	1,872 + 1,552	African American + European	exome + whole genome sequencing	NCBI build 37	Metabolon (MS)	70
(Lotta et al. 2016)[47]	Plasma	16,596	European	GWA	NCBI build 37	Biocrates (MS) + Metabolon (MS)	3
(Hu et al. 2017)[48]	Red blood cells + plasma	3,521 + 12,020	Chinese + European	GWA	NCBI build 36	gas chromatography + GC-MS	6
(Long et al. 2017)[49]	Serum	1,960	European	whole genome sequencing	NCBI build 38	Metabolon (MS)	644
(Davis et al. 2017)[50]	Serum	8,372	European	exome chip	NCBI build 37	Nightingale Health (NMR)	72
(Teslovich et al. 2018)[51]	Serum	8,545 + 2,591	European	GWA + exome chip	NCBI build 37	Nightingale Health (MS)	9 + 36 ratios

Study	***Biofluid***	***Sample (N)***	***Population***	***Study type***	***Genome build***	***Metabolomics platform(s)***	***Metabolites (N)***
(Feofanova et al. 2018)[52]	Serum	1,872 + 1,552	European + African American	whole exome + whole genome sequencing	NCBI build 37	Metabolon (MS)	102
(Kalsbeek et al. 2018)[53]	Red blood cells	2,374	European	GWA	NCBI build 37	gas chromatography	22 + 15 ratios
(de Oliveira Otto et al. 2018)[54]	Red blood cells + plasma	11,494	European	GWA	only rsids used	gas chromatography + GC-MS	5

Supplementary Table 2. Convergence of the GCTA results for the 4-variance component models for the 12 HMDB classes of metabolites of the Nightingale Health ^{1}H-NMR, UPLC-MS lipidomics, Leiden-^{1}H-NMR and Biocrates metabolomics platforms.

This table provides the number of metabolite loci (class-specific metabolite-SNP associations after clumping; see Supplementary Note 1 and Supplementary Figure 1) for each of the 12 HMDB classes available within our dataset, as well as the number of metabolites included in each of the 12 HMDB classes. For each HMDB class the number of metabolites for which the 4-variance component models did or did not converge is indicated. Additionally, for each HMDB class the HMDB super class is given.

Metabolite super class	Metabolite class	Number of SNPs in class-specific LDAK GRM	Number of Metabolites in Class	Number converged analyses	Number of failed analyses
Organic acids and derivatives	KetoAcids And Derivatives	20	2	0	2
	Hydroxy Acids And Derivatives	49	5	0	5
	Carboxylic Acids And Derivatives	400	44	43	1
Lipids and lipid-like molecules	Glycerolipids	32	47	5	42
	Sphingolipids	35	43	7	36
	Glycerophospholipids	85	145	104	41
	Steroids And Steroid Derivatives	108	7	3	4
	Lipoprotein	239	63	62	1
	FattyAcyls	260	11	11	0
Organic nitrogen compounds	Organonitrogen Compounds	30	1	0	1
Organic oxygen compounds	Organooxygen Compounds	51	9	1	8
Protein	Protein	80	3	0	3

Supplementary Table 3. Convergence of the GCTA results for the 4-variance component models for the 2 HMDB super classes of metabolites of the Nightingale Health ^{1}H-NMR, UPLC-MS lipidomics, Leiden-^{1}H-NMR and Biocrates metabolomics platforms.

This table provides the number of metabolite loci (class-specific metabolite-SNP associations after clumping; see Supplementary Note 1 and Supplementary Figure 1) for each of the 2 HMDB super classes available within our dataset, as well as the HMDB classes captured by the super classes and the number of metabolites included in each of the 2 HMDB super classes. For each HMDB super class the number of metabolites for which the 4-variance component models did or did not converge is indicated. Full model results can be found in Supplementary Data 3.

**Metabolite Superclass**	**Metabolite classes included in superclass**	**Number of SNPs in class-specific LDAK GRM**	**Number of Metabolites**	**Number converged analyses**	**Number of failed analyses**
Lipids and lipid-like molecules	Fatty Acyls	479	316	309	7
	Glycerolipids				
	Glycerophospholipids				
	lipoprotein				
	Sphingolipids				
	Steroids and steroid derivatives				
Organic acids and derivatives	Carboxylic acids and derivatives	397	53	52	1
	Hydroxy acids and derivatives				
	Keto acids and derivatives				
Organic nitrogen compounds	Organonitrogen Compounds	NA	1	NA	NA
Organic oxygen compounds	Organooxygen Compounds	NA	9	NA	NA
Protein	Protein	NA	3	NA	NA

Supplementary Table 4. categorization of the 53 organic acids into essential or non-essential amino acids.

Not all organic acids can be classified as either an essential or a non-essential amino acid as these are derivatives of amino acids, for these types of organic acids the 'type of amino acid' is blank. For full metabolite names see Supplementary Data 2.

metabolite	Type amino acid	metabolite	Type amino acid
Lac		Gly	Non-essential
Cit		Bet	
Ala	Non-essential	Cre	
His	Essential	Crea	
Ile	Essential	Pro	Non-essential
Leu	Essential	Tyr	Non-essential
Val	Essential	Phe	Essential
Tyr	Non-essential	For	
bOHBut		Arg	Non-essential
Crea		Gln	Non-essential
2bOHBut		Gly	Non-essential
Leu	Essential	His	Essential
Ile	Essential	Met	Essential
Val	Essential	Orn	
3bOHiBut		Phe	Essential
?KIV		Pro	Non-essential
3bOHBut		Ser	Non-essential
Ala	Non-essential	Thr	Essential
Ace		Trp	Essential
Met	Essential	Tyr	Non-essential
Glu	Non-essential	Val	Essential
Pyr		xLeu	Essential
Gln	Non-essential	AAA	
Cit			
Ca-EDTA			
Mg-EDTA			
DMG			
?KG			
Lys	Essential		
Orn			

Supplementary Table 5. Overview of covariates included in two-variance component GCTA analyses

Full model	Reduced model	Sparse model
First 10 genetic Dutch PCs	First 10 genetic Dutch PCs	First 10 genetic Dutch PCs
Genotyping method	Genotyping method	Genotyping method
Measurement batch*	Measurement batch*	Measurement batch*
Sex	Sex	Sex
Age at blood draw	Age at blood draw	Age at blood draw
BMI	BMI	
Smoking status	Smoking status	
Diabetes medication	Diabetes medication	
Anti-hypertensive medication	Anti-hypertensive medication	
Sex-hormones	Sex-hormones	
COPD medication		
Systemic hormones		
Anti-inflammatory medication		

* only included for the Biocrates and Nightingale Health ^{1}H-NMR platforms.

Supplementary Table 6. Summary of heritability estimates and standard errors (s.e.'s) of all two-variance component models for all metabolites.

Overview of the different models can be found in Supplementary Table 5.

**heritability**	**variable**	**full estimate**	**full s.e.**	**reduced estimate**	**reduced s.e.**	**sparse estimate**	**sparse s.e.**
h^2_{total}	mean	0.45	0.04	0.45	0.04	0.46	0.04
h^2_{total}	median	0.45	0.03	0.45	0.03	0.46	0.03
h^2_{total}	range	(0.12 - 0.73)	(0.02 - 0.07)	(0.12 - 0.73)	(0.02 - 0.07)	(0.11 - 0.74)	(0.02 - 0.07)
h^2_{SNP}	mean	0.06	0.24	0.07	0.24	0.08	0.24
h^2_{SNP}	median	0.08	0.23	0.08	0.23	0.10	0.23
h^2_{SNP}	range	(-0.64 - 0.79)	(0.11 - 0.36)	(-0.59 - 0.81)	(0.11 - 0.36)	(-0.57 - 0.83)	(0.11 - 0.35)

Supplementary References

1. Soininen, P., Kangas, A. J., Würtz, P., Suna, T. & Ala-Korpela, M. Quantitative Serum Nuclear Magnetic Resonance Metabolomics in Cardiovascular Epidemiology and Genetics. *Circ. Cardiovasc. Genet.* **8**, 192–206 (2015).

2. Würtz, P. *et al.* Quantitative Serum Nuclear Magnetic Resonance Metabolomics in Large-Scale Epidemiology: A Primer on -Omic Technology. *Am. J. Epidemiol.* **186**, 1–13 (2017).

3. Glauner, T., Zavitsanos, A. P. Electrospray operational parameters in TOF-MS. in *Comprehensive Analytical Chemistry volume 58 TOF-MS within food and environmental analysis* (ed. Barcelo, D.) 273–306 (Elsevier B.V., 2012).

4. Demirkan, A. *et al.* Insight in Genome-Wide Association of Metabolite Quantitative Traits by Exome Sequence Analyses. *PLoS Genet.* **11**, e1004835 (2015).

5. Römisch-Margl, W. *et al.* Procedure for tissue sample preparation and metabolite extraction for high-throughput targeted metabolomics. *Metabolomics* **8**, 133–142 (2012).

6. Goek, O. N. *et al.* Serum metabolite concentrations and decreased GFR in the general population. *Am. J. Kidney Dis.* **60**, 197–206 (2012).

7. Yang, J., Lee, S. H., Goddard, M. E. & Visscher, P. M. GCTA: A Tool for Genome-wide Complex Trait Analysis. *Am. J. Hum. Genet.* **88**, 76–82 (2011).

8. Speed, D., Hemani, G., Johnson, M. R. & Balding, D. J. Improved heritability estimation from genome-wide SNPs. *Am. J. Hum. Genet.* **91**, 1011–1021 (2012).

9. Speed, D., Cai, N., Johnson, M. R., Nejentsev, S. & Balding, D. J. Reevaluation of SNP heritability in complex human traits. *Nat. Genet.* **49**, 986–992 (2017).

10. Gieger, C. *et al.* Genetics meets metabolomics: a genome-wide association study of metabolite profiles in human serum. *PLoS Genet.* **4**, e1000282 (2008).

11. Illig, T. *et al.* A genome-wide perspective of genetic variation in human metabolism. *Nat. Genet.* **42**, 137–41 (2010).

12. Kettunen, J. *et al.* Genome-wide association study identifies multiple loci influencing human serum

metabolite levels. *Nat. Genet.* **44**, 269–276 (2012).

13. Draisma, H. H. M. *et al.* Genome-wide association study identifies novel genetic variants contributing to variation in blood metabolite levels. *Nat. Commun.* **6**, 7208 (2015).

14. Zaitlen, N. *et al.* Using Extended Genealogy to Estimate Components of Heritability for 23 Quantitative and Dichotomous Traits. *PLoS Genet.* **9**, (2013).

15. Nyholt, D. R. A simple correction for multiple testing for single-nucleotide polymorphisms in linkage disequilibrium with each other. *Am. J. Hum. Genet.* **74**, 765–9 (2004).

16. van Dongen, J. *et al.* Longitudinal weight differences, gene expression and blood biomarkers in BMI-discordant identical twins. *Int. J. Obes.* **39**, 899–909 (2015).

17. Abdellaoui, A. *et al.* Population structure, migration, and diversifying selection in the Netherlands. *Eur. J. Hum. Genet.* **21**, 1277–1285 (2013).

18. Aschard, H., Vilhjálmsson, B. J., Joshi, A. D., Price, A. L. & Kraft, P. Adjusting for heritable covariates can bias effect estimates in genome-wide association studies. *Am. J. Hum. Genet.* **96**, 329–339 (2015).

19. Kastenmüller, G., Raffler, J., Gieger, C. & Suhre, K. Genetics of human metabolism: an update. *Hum. Mol. Genet.* **24**, R93–R101 (2015).

20. Tanaka, T. *et al.* Genome-wide association study of plasma polyunsaturated fatty acids in the InCHIANTI Study. *PLoS Genet.* **5**, e1000338 (2009).

21. Hicks, A. a *et al.* Genetic determinants of circulating sphingolipid concentrations in European populations. *PLoS Genet.* **5**, e1000672 (2009).

22. Lemaitre, R. N. *et al.* Genetic loci associated with plasma phospholipid n-3 fatty acids: a meta-analysis of genome-wide association studies from the CHARGE Consortium. *PLoS Genet.* **7**, e1002193 (2011).

23. Suhre, K. *et al.* Human metabolic individuality in biomedical and pharmaceutical research. *Nature* **477**, 54–60 (2011).

24. Nicholson, G. *et al.* A genome-wide metabolic QTL analysis in Europeans implicates two loci shaped

by recent positive selection. *PLoS Genet.* **7**, e1002270 (2011).

25. Demirkan, A. *et al.* Genome-wide association study identifies novel loci associated with circulating phospho- and sphingolipid concentrations. *PLoS Genet.* **8**, e1002490 (2012).

26. Tukiainen, T. *et al.* Detailed metabolic and genetic characterization reveals new associations for 30 known lipid loci. *Hum. Mol. Genet.* **21**, 1444–55 (2012).

27. Krumsiek, J. *et al.* Mining the Unknown: A Systems Approach to Metabolite Identification Combining Genetic and Metabolic Information. *PLoS Genet.* **8**, (2012).

28. Wu, J. H. Y. *et al.* Genome-wide association study identifies novel loci associated with concentrations of four plasma phospholipid fatty acids in the de novo lipogenesis pathway: results from the Cohorts for Heart and Aging Research in Genomic Epidemiology (CHARGE) consortiu. *Circ. Cardiovasc. Genet.* **6**, 171–83 (2013).

29. Raffler, J. *et al.* Identification and MS assisted interpretation of genetically influenced NMR signals in human plasma. *Genome Med.* **5**, 13 (2013).

30. Hong, M. G. *et al.* A Genome-Wide Assessment of Variability in Human Serum Metabolism. *Hum. Mutat.* **34**, 515–524 (2013).

31. Xie, W. *et al.* Genetic Variants Associated With Glycine Metabolism and Their Role in Insulin Sensitivity and Type 2 Diabetes. *Diabetes* **62**, 2141–2150 (2013).

32. Rhee, E. P. *et al.* A genome-wide association study of the human metabolome in a community-based cohort. *Cell Metab.* **18**, 130–143 (2013).

33. Shin, S.-Y. *et al.* An atlas of genetic influences on human blood metabolites. *Nat. Genet.* **46**, 543–50 (2014).

34. Guan, W. *et al.* Genome-Wide association study of plasma n6 polyunsaturated fatty acids within the cohorts for heart and aging research in genomic epidemiology consortium. *Circ. Cardiovasc. Genet.* **7**, 321–331 (2014).

35. Mozaffarian, D. *et al.* Genetic loci associated with circulating phospholipid trans fatty acids: a meta-analysis of genome-wide association studies from the CHARGE Consortium. *Am. J. Clin. Nutr.* **101**, 398–406 (2015).

36. Lemaitre, R. N. *et al.* Genetic loci associated with circulating levels of very long-chain saturated fatty acids. *J. Lipid Res.* **56**, 176–184 (2015).

37. Yu, B. *et al.* Association of Rare Loss-Of-Function Alleles in HAL, Serum Histidine: Levels and Incident Coronary Heart Disease. *Circ. Cardiovasc. Genet.* **8**, 351–355 (2015).

38. Tintle, N. L. *et al.* A genome-wide association study of saturated, mono- and polyunsaturated red blood cell fatty acids in the Framingham Heart Offspring Study. *Prostaglandins Leukot. Essent. Fat. Acids* **94**, 65–72 (2015).

39. Hu, Y. *et al.* Genome-wide meta-analyses identify novel loci associated with n-3 and n-6 polyunsaturated fatty acid levels in chinese and european-ancestry populations. *Hum. Mol. Genet.* **25**, 1215–1224 (2015).

40. Hartiala, J. A. *et al.* Genome-wide association study and targeted metabolomics identifies sex-specific association of CPS1 with coronary artery disease. *Nat. Commun.* **7**, 1–10 (2016).

41. Kettunen, J. *et al.* Genome-wide study for circulating metabolites identifies 62 loci and reveals novel systemic effects of LPA. *Nat. Commun.* **7**, 11122 (2016).

42. Yet, I. *et al.* Genetic influences on metabolite levels: A comparison across metabolomic platforms. *PLoS One* **11**, (2016).

43. Yazdani, A., Yazdani, A., Liu, X. & Boerwinkle, E. Identification of Rare Variants in Metabolites of the Carnitine Pathway by Whole Genome Sequencing Analysis. *Genet. Epidemiol.* **40**, 486–491 (2016).

44. Fall, T. *et al.* Non-targeted metabolomics combined with genetic analyses identifies bile acid synthesis and phospholipid metabolism as being associated with incident type 2 diabetes. *Diabetologia* **59**, 2114–2124 (2016).

45. Rhee, E. P. *et al.* An exome array study of the plasma metabolome. *Nat. Commun.* **7**, 12360 (2016).

46. Yu, B. *et al.* Whole genome sequence analysis of serum amino acid levels. *Genome Biol.* **17**, 1–10 (2016).

47. Lotta, L. A. *et al.* Genetic Predisposition to an Impaired Metabolism of the Branched-Chain Amino Acids and Risk of Type 2 Diabetes: A Mendelian Randomisation Analysis. *PLOS Med.* **13**, e1002179

(2016).

48. Hu, Y. *et al.* Discovery and fine-mapping of loci associated with MUFAs through trans-ethnic meta-analysis in Chinese and European populations. *J. Lipid Res.* **58**, 974–981 (2017).

49. Long, T. *et al.* Whole-genome sequencing identifies common-to-rare variants associated with human blood metabolites. *Nat. Genet.* **49**, 568–578 (2017).

50. Davis, J. P. *et al.* Common, low-frequency, and rare genetic variants associated with lipoprotein subclasses and triglyceride measures in Finnish men from the METSIM study. *PLoS Genet.* **13**, 1–21 (2017).

51. Teslovich, T. M. *et al.* Identification of seven novel loci associated with amino acid levels using single-variant and gene-based tests in 8545 Finnish men from the METSIM study. *Hum. Mol. Genet.* **27**, 1664–1674 (2018).

52. Feofanova, E. V. *et al.* Sequence-Based Analysis of Lipid-Related Metabolites in a Multiethnic Study. *Genetics* **209**, 607–616 (2018).

53. Kalsbeek, A. *et al.* A genome-wide association study of red-blood cell fatty acids and ratios incorporating dietary covariates: Framingham heart study offspring cohort. *PLoS One* **13**, 1–16 (2018).

54. De Oliveira Otto, M. C. *et al.* Genome-wide association meta-analysis of circulating odd-numbered chain saturated fatty acids: Results from the CHARGE Consortium. *PLoS One* **13**, 1–12 (2018).
