# Supplementary material for: Heritability estimates for 361 blood metabolites across 40 genome-wide association studies
Source: Nat Commun. 2020 Jan 7;11:39. doi: 10.1038/s41467-019-13770-6 (PMC6946682; doi:10.1038/s41467-019-13770-6)
Supplement: Supplementary file 18 — Reporting Summary [file 41467_2019_13770_MOESM18_ESM.pdf]

nature research

Corresponding author(s): Fiona A. Hagenbeek, Michel G. Nivard, Dorret I. Boomsma

Last updated by author(s): Nov 20, 2019

Reporting Summary

Nature Research wishes to improve the reproducibility of the work that we publish. This form provides structure for consistency and transparency in reporting. For further information on Nature Research policies, see Authors & Referees and the Editorial Policy Checklist.

Statistics

For all statistical analyses, confirm that the following items are present in the figure legend, table legend, main text, or Methods section.

n/a	Confirmed	
☐	☒	The exact sample size (*n*) for each experimental group/condition, given as a discrete number and unit of measurement
☐	☒	A statement on whether measurements were taken from distinct samples or whether the same sample was measured repeatedly
☐	☒	The statistical test(s) used AND whether they are one- or two-sided<br>*Only common tests should be described solely by name; describe more complex techniques in the Methods section.*
☐	☒	A description of all covariates tested
☐	☒	A description of any assumptions or corrections, such as tests of normality and adjustment for multiple comparisons
☐	☒	A full description of the statistical parameters including central tendency (e.g. means) or other basic estimates (e.g. regression coefficient) AND variation (e.g. standard deviation) or associated estimates of uncertainty (e.g. confidence intervals)
☐	☒	For null hypothesis testing, the test statistic (e.g. *F*, *t*, *r*) with confidence intervals, effect sizes, degrees of freedom and *P* value noted<br>*Give P values as exact values whenever suitable.*
☒	☐	For Bayesian analysis, information on the choice of priors and Markov chain Monte Carlo settings
☒	☐	For hierarchical and complex designs, identification of the appropriate level for tests and full reporting of outcomes
☐	☒	Estimates of effect sizes (e.g. Cohen's *d*, Pearson's *r*), indicating how they were calculated

Our web collection on statistics for biologists contains articles on many of the points above.

Software and code

Policy information about availability of computer code

Data collection	Agilent MassHunter Quantitative Analysis software (Agilent, Version B.04.00); PLINK 1.9; KING; MACH-ADMIX; PERL based "HRC or 1000G Imputation preparation and checking" tool (version 4.2.5); EAGLE; SHAPEIT; Minimac3; SMARTPCA; R version 3.5.1
Data analysis	GCTA version 1.91.7, LDAK version 4.9 and R version 3.5.1 for 'metafor' package (version 2.0-0), MASS package (version 7.3-50) and gee package (version 4.13-19)

For manuscripts utilizing custom algorithms or software that are central to the research but not yet described in published literature, software must be made available to editors/reviewers. We strongly encourage code deposition in a community repository (e.g. GitHub). See the Nature Research guidelines for submitting code & software for further information.

Data

Policy information about availability of data

All manuscripts must include a data availability statement. This statement should provide the following information, where applicable:

- Accession codes, unique identifiers, or web links for publicly available datasets
- A list of figures that have associated raw data
- A description of any restrictions on data availability

The curated list of all published metabolite-SNP associations is included in Supplementary Data 1 and is publicly available through the BBMRI – omics atlas (http://bbmri.researchlumc.nl/atlas/#data). All information on the metabolites in this study are in Supplementary Data 2; with full summary statistics for the four-variance component models included in Supplementary Data 3. The Nightingale Health metabolomics data may be requested through BBMRI-NL (https://www.bbmri.nl/Omics-metabolomics). All (other) data may be accessed, upon approval of the data access committee, through the Netherlands Twin Register (ntr.fgb@vu.nl). A reporting summary for this Article is available as Supplementary Information file.

Field-specific reporting

Please select the one below that is the best fit for your research. If you are not sure, read the appropriate sections before making your selection.

☐ Life sciences ☒ Behavioural & social sciences ☐ Ecological, evolutionary & environmental sciences

For a reference copy of the document with all sections, see nature.com/documents/nr-reporting-summary-flat.pdf

Behavioural & social sciences study design

All studies must disclose on these points even when the disclosure is negative.

Study description	The final number of participants included in the study was 5,117, with platform specific sample size ranging from 1,448 to 4,227 individuals from 946 to 2,179 families. Characteristics for the individuals included in the analyses can be found in Table 2.
Research sample	At the Netherlands Twin Register (NTR) metabolomics data for twins and family members as measured in blood samples were available for 6,011 individuals of whom 5,667 were genotyped. The blood samples for the four metabolomics experiments described in this study were mainly collected in participants of the NTR biobank project.
Sampling strategy	For NTR biobank individuals of 18 years and over who previously completed one or more surveys as part of the longitudinal NTR study and wereregistered as active participants with the NTR were selected for the study.
Data collection	Blood samples were collected after a minimum of two hours of fasting (1.3%), with the majority of the samples collected after overnight fasting (98.7%). Fertile women were bled in their pill-free week or on day 2-4 of their menstrual cycle.<br>Metabolie profiling was done using the Nightingale Health 1H-NMR platform (Nightingale Health Ltd., Helsinki, Finland), a lipidomics platform (Leiden/Amsterdam Center for Drug Research, Leiden University, the Netherlands) using ultra-high performance liquid chromatograph coupled to an Electrospray Ionization Quadruple Time-of-Flight high resolution mass spectrometer (UPLC-ESI-Q-TOF; Agilent 6530, San Jose, CA, USA), the Leiden 1H-NMR platform (for small metabolites; 600 MHz Bruker Advance II spectrometer [Bruker BioSpin, Karlsruhe, Germany]) and the Biocrates Absolute-IDQ p150 (Biocrates Life Sciences AG, Innsbruck, Austria) platform. Details with regards to these platforms may be found in the methods section.<br>Genotyping in the NTR was performed using 6 different genotyping arrays (Affymetrix 6.0, Perlegen-Affymetrix, Illumina Human Quad Bead 660, Affymetrix Axiom, Illumnia GSA and Illumina Omni Express 1M), as well as sequence data from the Netherlands reference genome project GONL (BGI full sequence at 12x). Full details about genotyping and imputation may be found in the methods section.
Timing	NTR biobank collection ran between January 2004 and July 2008
Data exclusions	participants were excluded if they were not of Dutch ancestry, were on lipid-lowering medication at the time of blood draw or if they had not adhered to the fasting protocol. The exact number of exclusions is listed in Supplementary Table 7.
Non-participation	Of the individuals invited for participation in NTR biobank (14,093 participants, 64% of adult NTR participants): 96 individuals had died, 54 lived abroad, 2,190 individuals were not reached by phone, 193 had problems deciding to participate and 3,434 did not want to participate. Furthermore, for 453 individuals who agreed to participate an appointment was difficult to make.
Randomization	Participants were not allocated into experimental groups.

Reporting for specific materials, systems and methods

We require information from authors about some types of materials, experimental systems and methods used in many studies. Here, indicate whether each material, system or method listed is relevant to your study. If you are not sure if a list item applies to your research, read the appropriate section before selecting a response.

Materials & experimental systems

n/a	Involved in the study	
☒	☐	Antibodies
☒	☐	Eukaryotic cell lines
☒	☐	Palaeontology
☒	☐	Animals and other organisms
☐	☒	Human research participants
☒	☐	Clinical data

Methods

n/a	Involved in the study	
☒	☐	ChIP-seq
☒	☐	Flow cytometry
☒	☐	MRI-based neuroimaging

Human research participants

Policy information about studies involving human research participants

Population characteristics	See above

Recruitment	All participants in this study are part of the NTR longitudinal study. The majority of families registered with the NTR were recruited when the twins were adolescents or young adults through City Council registration systems in 1990–91 and in 1992–93. Since 1993, adult twins are recruited through a variety of other approaches, such as advertisements (see Boomsma et al., 2002, 2006).
Ethics oversight	the Central Ethics Committee on Research Involving Human Subjects of the VU University Medical Centre, Amsterdam, an Institutional Review Board certified by the U.S. Office of Human Research Protections (IRB number IRB00002991 under Federal-wide Assurance- FWA00017598; IRB/institute codes, NTR 03-180 and EMIF-AD 2014.210).

Note that full information on the approval of the study protocol must also be provided in the manuscript.
