# Supplementary material for: Heritability estimates for 361 blood metabolites across 40 genome-wide association studies
Source: Nat Commun. 2020 Jan 7;11:39. doi: 10.1038/s41467-019-13770-6 (PMC6946682; doi:10.1038/s41467-019-13770-6)
Supplement: Supplementary file 19 — Description of Additional Supplementary Files [file 41467_2019_13770_MOESM19_ESM.pdf]

Title: Supplementary Data 1.
Description: The metabolite-SNP or metabolite ratio-SNP associations as identified by 40 GWA and (exome-) sequencing studies conducted in the period of November 2008 to October 2018.
The complete references for all 40 studies can be found in Supplementary Table 1 and additional information about the curation of this list is described in the Method section of the manuscript.

Supplementary Data 2.
Description: Full biochemical metabolite names and untransformed quartile values of all 427 metabolites that are measured on the Nightingale Health ^{1}H-NMR, UPLC-MS Lipidomics, Leiden ^{1}H-NMR or Biocrates metabolomics platforms and survived QC and preprocessing.

Title: Supplementary Data 3.
Description: Four-variance component GCTA results for the Nightingale Health ^{1}H-NMR, UPLC-MS lipidomics, Leiden ^{1}H-NMR and Biocrates metabolomics platforms.
In this table the estimates and the s.e.'s of each of the four variance components (V(G1) to V(G4)) and each of the seven heritabilities are provided. Here V(G1) is equivalent to h^2_g, the additive genetic effects-based on genome-wide SNPs. V(G2) denotes the pedigree-based additive genetic effects (h^2_{ped}). V(G3) is equivalent to $h^2_{Class\text{-}hits}$, these estimates denote the heritability of the metabolite-class specific loci. V(G4)is equivalent to $h^2_{Notclass\text{-}hits}$, these estimates denote the heritability of the 'non-class' metabolite loci. The sum of V(G3) and V(G4) gives the total heritability of metabolite loci ($h^2_{metabolite\text{-}hits}$), the sum of V(G1), V(G3)and V(G4) gives the SNP-based heritability (h^2_{SNP}) and the sum of all four variance components gives the twin-family heritability estimate (h^2_{total}; see Figure 1). Failed[i] indicate that results for this metabolite could not be obtained as GCTA did not converge after 100 iterations after bending of the variance-covariance matrix and Failed[b] indicates bending of the variance-covariance matrix was not possible. Full names for all metabolites can be found in Supplementary Data 2.

Title: Supplementary Data 4.
Description: Cross-platform comparison of overlapping metabolites.
This table lists the Spearman's Rho phenotypic correlations and heritability estimates of the metabolites included on more than one platform. Here the metabolite names are preceded by the abbreviation of the platform they were measured on, with "Night" indicating this metabolite was measured on the Nightingale Health platform, "Lip" for the UPLC-MS lipidomics platform, "Leiden" for the Leiden ^{1}H-NMR platform and "Bioc" for the Biocrates platform. The standard errors as listed here are the adjusted standard errors (see methods).

Title: Supplementary Data 5.
Description: Multivariate mixed-effect meta-regression models for the factors influencing metabolite heritability corrected for metabolomic platform effects.
Original p-values for the model fit are given as wel as the FDR corrected p-values (see Methods).

Significant p-values ($p < 0.05$) are given in **bold**. [§]PCaa = phosphatidylcholines diacyl; PCae = phosphatidylcholine acyl-alkyl. [¥]TGs = triglycerides; not adjusted for platform as these have only been included on the UPLC-MS lipidomics platform

Title: Supplementary Data 6.
Description: Univariate mixed-effect meta-regression models for the factors influencing metabolite heritability.
Original p-values for the model fit are given as well as the FDR corrected p-values (see Methods). [§] PCaa = phosphatidylcholines diacyl; PCae = phosphatidylcholine acyl-alkyl. [¥]TGs = triglycerides

Title: Supplementary Data 7.
Description: Overview of the number of participants for each of the four metabolomics platforms prior to participant exclusions and the number of participants to be excluded.
The number of participants (*N*) with metabolomics data is given for each subset of data for each of the four metabolomics platforms, indicating in which measurement batch this data was included and as part of what dataset the data was collected. Here "BB1" indicates the data was collected during the first wave of the NTR Biobank project, "BB2" indicates the data was collected during the second wave of NTR Biobank and "EMIF-AD" indicates the data was collected as part of the EMIF-AD project. For each subset the number of participants with overlapping metabolomics and genotypic data, the mean and standard deviation (*SD*) of the age at blood draw, the percentage of females, the percentage of twins, the mean and SD of the body-mass-index (BMI) and of the total cholesterol, low-density lipoprotein (LDL) cholesterol and high-density lipoprotein (HDL) cholesterol levels are given. Finally, this table also lists the number of individuals to be excluded because they are not of Dutch origin, were on lipid-lowering medication at the time of blood draw or had not adhered to the fasting protocol.

Title: Supplementary Data 8.
Description: Number of overlapping individuals across the different subsets for the four metabolomics platforms.
On the diagonal the number of participants with metabolomics data in each dataset is given, the upper triangle off-diagonal values give the number of overlapping individuals with metabolomics data across all data sets. "BB1" denotes data as collected during the first wave of the NTR biobank, "BB2"denotes data as collected during the second wave of NTR biobank, "b1" denotes the first measurement batch, "b2"denotes the second measurement batch and "b3" denotes the third measurement batch.

Title: Supplementary Data 9.
Description: Sample overlap for all 427 metabolites across all four metabolomics platforms
This tables gives an overlap of the samples between each pair of metabolites, both within and cross platform, where "Night" indicating this metabolite was measured on the Nightingale Health

platform, "Lip" for the UPLC-MS lipidomics platform, "Leiden" for the Leiden ^{1}H-NMR platform and "Bioc" for the Biocrates platform.

Title: Supplementary Data 10.
Description: Pairwise phenotypic correlations of all 361 metabolites for which heritability estimates were successfully obtained.
Phenotypic correlations were calculated using Spearman's Rho. The platform on which each metabolite has been measured has been indicated by a prefix, where "Night" indicates the Nightingale Health platform, "Lip" indicates the UPLC-MS lipidomics platform, "Leiden" indicates the Leiden ^{1}H-NMR platform and "Bioc" indicates the Biocrates platform.

Title: Supplementary Data 11.
Description: Matrix combining the Sample overlap and phenotypic correlations for all 361 successfully analyzed metabolites across all four metabolomics platforms
This tables gives an overlap of the samples and the phenotypic correlation between each pair of metabolites, both within and cross platform, where "Night" indicating this metabolite was measured on the Nightingale Health platform, "Lip" for the UPLC-MS lipidomics platform, "Leiden" for the Leiden ^{1}H-NMR platform and "Bioc" for the Biocrates platform. This matrix includes the sample size of the metabolite on the diagonal, with the off-diagonal computed by N1,2/√(n1* n2)*r Where N1,2 is the sample overlap between the metabolites, N1 is the sample size of metabolite one, N2 is the sample size of metabolite two and r is the phenotypic correlation between the metabolites as calculated with Spearman's Rho.

Title: Supplementary Data 12.
Description: Results power analysis, using the GCTA power calculator, to compare the clumped LDAK GRMs with the not-clumped LDAK genetic relatedness matrices (GRM). The Type I error rate, α, has been set to 0.05 for all power calculations. We've calculated the power assuming the true SNP heritability (h^2_{SNP}) per GRM is 0.20, 0.10 or 0.05. The variance of the SNP-derived genetic relationships is based on the variance of the off-diagonal elements of each GRM. The power calculates provides us with the standard error (SE) of h^2_{SNP}, the non-centrality parameter (NCP) of the chi-squared test statistic ($h^4/(SE)^2$) and power (probability of detecting $h^2_{SNP} > 0$). Explanations of the different GRMs can be found in the Methods, and in Supplementary Figure 1.

Title: Supplementary Data 13. Results power analysis, using the GCTA power calculator, to compare
Description: the clumped LDAK GRMs with the clumped GCTA genetic relatedness matrices (GRM). The Type I error rate, α, has been set to 0.05 for all power calculations. We've calculated the power assuming the true SNP heritability (h^2_{SNP}) per GRM is 0.20, 0.10 or 0.05. The variance of the SNP-derived genetic relationships is based on the variance of the off-diagonal elements of each GRM. The power calculates provides us with the standard error (SE) of h^2_{SNP}, the non-centrality parameter

(NCP) of the chi-squared test statistic ($h^4/(SE)^2$) and power (probability of detecting $h^2_{SNP} > 0$). Explanations of the different GRMs can be found in the Methods, and in Supplementary Figure 1.

Title: Supplementary Data 14.
Description: GEE results for all metabolites of the Nightingale Health ^{1}H-NMR, UPLC-MS lipidomics, Leiden ^{1}H-NMR and Biocrates metabolomics platforms.
P-values in **bold** are significant after correcting for multiple testing, corrected for the correlation among the metabolites of the four platforms (p =< 0.0005). Full names and biochemical class for all metabolites can be found in Supplementary Data 2.

Title: Supplementary Data 15.
Description: two-variance component GCTA results for the Nightingale Health ^{1}H-NMR, UPLC-MS lipidomics, Leiden ^{1}H-NMR and Biocrates metabolomics platform
The two-variance component models, including narrow-sense heritability and SNP heritability for the full, reduced and sparse models. Overview of the different models can be found in Supplementary Note 4 and Supplementary Table 5. Full names and biochemical class for all metabolites can be found in Supplementary Data 2.
